# Systemic inflammatory biomarkers as prognostic tools in patients with gastroesophageal adenocarcinoma

**DOI:** 10.1007/s00432-023-05424-4

**Published:** 2023-09-26

**Authors:** Hannah C. Puhr, Clemens C. Weirauch, Flora Selimi, Karin Oberreiter, Martin A. Dieterle, Gerd Jomrich, Sebastian F. Schoppmann, Gerald W. Prager, Anna S. Berghoff, Matthias Preusser, Aysegül Ilhan-Mutlu

**Affiliations:** 1 Division of Oncology, Department of Medicine I, Medical University of Vienna, Waehringer Guertel 18-20, 1090 Vienna, Austria; 2 Christian Doppler Laboratory for Personalized Immunotherapy, Medical University of Vienna, Waehringer Guertel 18-20, 1090 Vienna, Austria; 3 Department of Surgery, Medical University of Vienna, Waehringer Guertel 18-20, 1090 Vienna, Vienna, Austria

**Keywords:** Gastric cancer, Oesophageal cancer, Inflammation, Prognosis

## Abstract

**Purpose:**

Gastroesophageal adenocarcinoma is associated with poor prognosis, even in resectable stages. Systemic inflammation plays a key role in cancer progression. Yet, information on prognostic values of systemic inflammatory parameters in European cohorts is scarce.

**Methods:**

We analysed systemic inflammatory biomarkers (neutrophil-to-lymphocyte ratio (NLR), leucocyte-to-lymphocyte ratio (LLR), platelet-to-lymphocyte ratio (PLR), systemic inflammation response index (SIRI) and modified Glasgow Prognostic Score (mGPS)) at the time of cancer diagnosis and their association with overall survival (OS) in patients with gastroesophageal adenocarcinoma treated at the Medical University of Vienna between 1990 and 2020.

**Results:**

In this analysis of 769 patients with gastroesophageal adenocarcinoma, higher mGPS (0–2) scores were associated with shorter OS in the overall cohort (24.9 versus 11.9 versus 7.6 months; HR 1.74, 95% CI 1.549–1.056; *p* < 0.001), in locally advanced (31.1 versus 19.8 versus 13.9 months, HR 1.561, 95% CI 1.274–1.912; *p* < 0.001) and in advanced/metastatic settings (12.3 versus 7.3 versus 5.8 months; HR 1.377, 95% CI 1.777–1.611; *p* < 0.001). In multivariate analyses, the association of mGPS with the OS stayed statistically significant in the locally advanced cohort (HR 1.397, 95% CI 1.068–1.828; *p* = 0.015), whereas NLR, LLR, PLR and SIRI did not. mGPS was associated with more advanced stages (*p* < 0.001) and weight loss (*p* = 0.002).

**Conclusion:**

mGPS poses a feasible prognostic tool in patients with locally advanced gastroesophageal cancer.

**Supplementary Information:**

The online version contains supplementary material available at 10.1007/s00432-023-05424-4.

## Introduction

Gastroesophageal cancer represents a major global disease burden with high mortality rates (Sung et al. 2021). Although recent advances in treatment strategies have improved overall survival, prognosis remains poor, even in resectable settings (Lordick et al. 2022; Obermannová et al. 2022). Thus, novel prognostic markers are warranted to improve patient management(Dhakras et al. 2020).

The rise of checkpoint inhibition did not only lead to several practices—changing drug approvals, but also raised awareness of local as well as systemic inflammatory parameters as predictive and prognostic parameters in several oncological entities including gastroesophageal cancers (Moehler et al. 2021; Shitara et al. 2022; Li et al. 2021). Systemic inflammatory biomarkers, including leucocyte ratios, such as neutrophil–lymphocyte ratio (NLR), lymphocyte–leucocyte ratio (LLR), platelet–lymphocyte ratio (PLR) and the systemic inflammation response index (SIRI; neutrophils*monocytes/lymphocytes) as well as the C-reactive protein (CRP) and albumin-based modified Glasgow prognostic score (mGPS), propose potential association with treatment outcome (Laird et al. 2013; Proctor et al. 2011). Retrospective analyses in Asian cohorts showed that systemic inflammation might be an important prognostic factor independent of tumour stage and treatment regimen (Matsumoto et al. 2023; Liu et al. 2021; Huang et al. 2020). However, large real-world investigations of Western cohorts comprising all tumour stages are lacking as existing data focus largely on prediction of surgical as well as pathological outcomes and do not investigate inflammatory markers in association with other known prognostic factors in multivariate settings (Kim et al. 2020; Schietroma et al. 2022; Nechita et al. 2022; Pikuła et al. 2022; Jomrich et al. 2021; Petrillo et al. 2018). As tumour biology, biomarker expression, treatment response and survival differ between Asian and Western cohorts (Rha et al. 2022; Zhang et al. 2022b), the aim of this analysis was to investigate systemic inflammatory processes at the time of first cancer diagnosis as prognostic markers in a large European cohort of gastroesophageal cancer patients.

## Methods

### Patient recruitment

Adult patients with gastroesophageal adenocarcinoma diagnosed and/or treated at the Department of Medicine I – Division of Oncology at the Medical University of Vienna between 01.01.1990 and 31.12.2020 with available laboratory parameters were included in this analysis independent of stage and tumour location. In order to reduce selection bias and improve representability, patients with isolated missing values were not excluded from this analysis. Clinical data including laboratory parameters were retrieved by structured electronical chart review. Disease was pathologically confirmed by expert pathologists. Analyses of human epidermal growth factor receptor 2 (HER2) and combined positive score (CPS) for PD-L1 testing have been routinely performed in metastatic settings since 2012 and 2018, respectively. *Helicobacter pylori* screening is not routinely performed; however, when infection is evident in microscopic specimen, it is reported.

Treatment strategies were reviewed by an interdisciplinary tumour board and patients received the best available treatment according to current treatment guidelines at the time of diagnosis.

Death was registered according to hospital chart data and/or public data provided by Statistik Austria. Patients without registered date of death at the time of data cut-off were censored at the date that they were last known to be alive. Overall survival (OS) was defined as the time from initial cancer diagnosis to patient’s death or last follow-up date.

In addition to analysis of the overall cohort, subgroups based on staging subgroups were performed to further investigate prognostic impact of systemic inflammatory parameters. Patients were divided into localised (stage 1), locally advanced (stage 2 and stage 3) and advanced/metastatic (stage 4) subgroups based on the latest (8th) edition of UICC TNM staging classification. This ensured minimization of potential bias due to improved diagnostic procedures over time and is in line with application of treatment strategies. Patient data were collected at the time of first diagnosis of gastroesophageal cancer, even if the patient experienced cancer recurrence throughout their history. Patients were only recorded once, also when they received multiple gastroesophageal tumours throughout their history. -All procedures were performed in accordance with the ethical standards of the responsible committee on human experimentation and with the Helsinki Declaration of 1964 and latter versions. The study was approved by the ethics committee of the Medical University of Vienna (reference number: 1600/2021).

### Laboratory parameters

Systemic laboratory parameters included total blood count including platelets (PLT), white blood cells/leucocytes (WBC), neutrophils, lymphocytes, monocytes, eosinophils, basophils as well as C-reactive protein (CRP) and albumin. Normal limits for laboratory values were PLT 150–350 G/l, WBC 4.0–10.0 G/l, neutrophils 2.0–7.5 G/l, lymphocytes 1.0–4.0 G/l, monocytes 0.0–1.2 G/l, eosinophils 0.0–0.4 G/l, basophils 0.0–0.1 G/l, CRP < 0.5 mg/dl and albumin ≥ 35 g/L. Other units were recalculated and transferred to the appropriate units, respectively.

Laboratory parameters were described as within, below and above normal limit according to aforementioned limits. In addition, systemic inflammation ratios (neutrophil–lymphocyte ratio (NLR), lymphocyte–leucocyte ratio (LLR), platelet–lymphocyte ratio (PLR), systemic inflammation response index (SIRI)) and the modified Glasgow Prognostic Score (mGPS) (Proctor et al. 2011) were calculated.

NLR was defined as simple ratio between absolute neutrophil and absolute lymphocyte counts, LLR as simple ratio between absolute lymphocyte count and absolute leucocyte count and PLR as simple ratio between absolute platelet count and absolute lymphocyte. SIRI was defined as absolute neutrophil count multiplied by absolute monocyte count divided by absolute lymphocyte count. mGPS was scored 0 when CRP was < 1.0 mg/dl, 1 when CRP was > 1.0 mg/dl and albumin was ≥ 35 g/L, and scored 2 when CRP was > 1.0 mg/dl and albumin was < 35 g/L. For further evaluation, patients were divided into high and low groups according to median levels of NLR, LLR, PLR and SIRI (≤ median or > median) respective to the subgroup.

Blood taken for the analysis was performed regularly before administration of therapy and/or at diagnosis and evaluated retrospectively with structured electronical chart review. Laboratory parameters were classified as being conducted at first diagnosis, when the blood draw took place at the time of histopathological first cancer diagnosis or within 1 month before or after this date. When blood was taken within 1 month before or 1 month after histopathological first cancer diagnosis date, it was only considered for this analysis when no tumour-specific therapy (i.e. surgery, systemic therapy, radiation therapy) had taken place in this time frame. When multiple laboratory analyses were available, the one which was closest to histopathological first diagnosis was considered for this analysis.

As more severe disease stages as well as tumour location, sarcopenia, helicobacter infection, nicotine and alcohol abuse have been associated with higher systemic inflammatory parameters, associations between NLR, LLR, PLR and mGPS with cancer staging subgroups (localised, locally advanced, advanced or metastatic) as well as weight loss and BMI were performed.

### Statistical analysis

Upon retrospective data collection, results were entered into the statistical package for the social sciences (SPSS) 20.0 software (SPSS Inc., Chicago, IL, USA).

Descriptive analyses of clinical data were performed by mean (SD) for metric and median for nominal data. Calculations for survival differences were performed with log-rank test for nominal variables and with Cox regression for metric variables. Visualisation of survival analyses was performed with Kaplan–Meier product. Cox proportional hazard model was used for multivariate analysis, which included all variables with statistically significant results in univariate analysis. For further association analyses between staging subgroups, weight loss and BMI with systemic inflammatory ratios, the chi-square test and Kruskal–Wallis test were performed as appropriate. *P*-values < 0.05 were considered to indicate statistical significance. Due to the hypothesis generating design of the studies, no correction for multiple testing was applied unless specified otherwise (Bender and Lange 2001).

## Results

### Patient and tumour characteristics

In this analysis, we included 769 patients with gastroesophageal adenocarcinoma. 68% of patients were male. The mean age was 63.9 years (SD 12.2). 49% of tumours were located in the stomach, 37% in the gastroesophageal junction area and 14% in the oesophagus. Concerning staging, 101 (13%) patients had a localised disease (UICC stage 1), 361 (47%) patients were categorised as locally advanced disease (stage 2: *n* = 125, stage 3: *n* = 236) and 307 (40%) patients had a primary advanced or metastatic disease (UICC stage 4) at initial cancer diagnosis. Patient and tumour characteristics for localised, locally advanced and advanced or metastatic subgroups are available in Supplementary tables 1 + 2.

At the time of data cut-off (November 1, 2022), 620 patients (81%) of the population were already deceased. The mean overall survival (OS) of the population was 38.1 months (SD 51.9). Median OS was statistically significantly associated with tumour stage with timespan decreasing with severity of stage (localised: median 102.2 months, 95% CI 72.1–132.3; locally advanced: 24.3 months, 95% CI 19.3–29.3; advanced or metastatic: 9.8 months, 95% CI 8.1–11.5; *p* < 0.001). Further classification of locally advanced adenocarcinomas showed that median OS was longer in stage 2 than in stage 3 patients (38.4 months, 95% CI 24.4–52.4 versus 21.4 months, 95% CI 18.1–24.7; *p* = 0.008).

Age was also associated with OS in the overall cohort (HR 1.008; 95% CI 1.001–1.015; *p* = 0.018) as well as in subgroups (localised: HR 1.046, 95% CI 1.021–1.073; *p* < 0.00; locally advanced: HR 1.012, 95% CI 1.001–1.023; *p* = 0.032; advanced or metastatic: HR 1.010; 95% CI 1.000–1.020; *p* = 0.041).

Further patient, tumour characteristics and their association with the OS in the overall cohort as well as in subgroups can be seen in Supplementary Tables 1 and 2.

Concerning treatment regimens, 494 patients (64%) had a surgical resection of the primary tumour [localised: 94 (93%), locally advanced: 318 (88%), primary advanced or metastatic: 81 (26%)]. 76 patients (10%) received radio(chemo)therapy for the primary tumour, 21 patients received definitive radiochemotherapy without surgical resection. In localised setting, 11 patients (11%) received systemic chemotherapy (neoadjuvant/perioperative: 1, adjuvant: 6, definitive radiochemotherapy: 4). In locally advanced setting, 178 patients (49%) received perioperative chemotherapy (neoadjuvant/perioperative: 132, adjuvant: 33, definitive: 13). In advanced or metastatic setting, 237 patients (77%) received chemotherapy (neoadjuvant/perioperative: 4, adjuvant: 2, palliative: 227, definitive radiochemotherapy: 4; patients who received neoadjuvant/perioperative, adjuvant chemotherapy and definitive radiochemotherapy had oligometastatic disease).

Patients who received tumour-specific treatment had longer OS than patients who received best supportive care in the overall cohort as well as in locally advanced and primary metastatic settings (overall cohort (*n* = 721 vs 48): 20.1 (17.9–22.3) months, *p* < 0.001 vs 3.4 (95% CI 1.7–5.1) vs; localised setting (*n* = 96 vs 5): 102.2 (72.0–132.4) vs 65.4 (n.a.) months, *p* = 0.807; locally advanced (*n* = 356 vs 5): 25.1 (20.0–30.2) versus 10.1 (2.5–17.7) months, *p* < 0.001; metastatic (*n* = 269 vs 38: 11.3 (9.8–12.8) vs 3.2 (2.0–4.4) months, *p* < 0.001).

### Systemic inflammation

Laboratory parameters for the overall cohort and subgroups are available in Supplementary Table 3.

Neutrophil–lymphocyte ratio (NLR) was available in 598 patients, lymphocyte–leucocyte ratio (LLR) in 594 patients, platelet–lymphocyte ratio (PLR) in 567 patients and systemic inflammation response index (SIRI) in 597 patients. Mean and median values for inflammatory ratios are available in Table 1.

**Table 1:** Systemic inflammatory ratios in a cohort of 769 patients with gastroesophageal adenocarcinoma

Variable	Overall cohort			Localized			Locally advanced			Advanced or metastatic		
	*n*	Mean (SD)	Median	*n*	Mean (SD)	Median	*n*	Mean (SD)	Median	*n*	Mean (SD)	Median
NLR	598	4.2 (3.3)	3.3	94	3.5 (2.2)	2.9	299	4.0 (3.3)	3.2	205	4.7 (3.6)	3.9
LLR	594	5.7 (3.5)	4.8	94	5.0 (2.4)	4.4	297	5.5 (3.5)	4.7	203	6.4 (3.9)	5.6
PLR	567	208.4 (144.3)	180.9	88	171.0 (84.9)	160.7	278	194.6 (111.4)	170.8	201	244.0 (191.0)	200.0
SIRI	597	2.6 (2.4)	1.8	94	2.2 (2.1)	1.5	299	2.3 (2.3)	1.6	204	3.2 (2.7)	2.5

Abbreviations: *NLR* neutrophil–lymphocyte ratio, *LLR* lymphocyte–leucocyte ratio, *PLR* platelet–lymphocyte ratio, *SD* standard deviation, *HR* hazard ratio, *CI* confidential interval

In 688 patients, mGPS was available, with 456 score 0 (59%), 152 score 1 (20%) and 80 score 2 (10%). mGPS for stage-based subgroups are available in Table 2.

**Table 2:** Modified Glasgow prognostic score (mGPS), neutrophil–lymphocyte ratio (NLR), lymphocyte–leucocyte ratio (LLR), platelet–lymphocyte ratio (PLR), systemic inflammation response index (SIRI) in a cohort of 769 patients with gastroesophageal adenocarcinoma and their association with the overall survival (log-rank test)

Variable	Overall cohort			Localized			Locally advanced			Advanced or metastatic		
	*n* (%)	OS in months (95%CI)	*p*	*n* (%)	OS in months (95%CI)	*p*	*n* (%)	OS in months (95%CI)	*p*	*n* (%)	OS in months (95%CI)	*p*
mGPS			** < 0.001**			0.174			** < 0.001**			** < 0.001**
Score 0	456 (59%)	24.9 (20.4–29.4)		79 (78%)	110.8 (85.4–136.2)		233 (64%)	31.1 (21.8–40.4)		144 (47%)	12.3 (10.2–14.4)	
Score 1	152 (20%)	11.9 (8.7–15.1)		8 (8%)	65.0 (0–135.1)		64 (18%)	19.8 (11.9–27.7)		80 (26%)	7.3 (5.8–8.8)	
Score 2	80 (10%)	7.6 (4.8–10.4)		5 (5%)	26.2 (0–124.1)		20 (6%)	13.9 (8.6–19.2)		55 (18%)	5.8 (3.1–8.5)	
missing	81 (11%)			9 (9%)			44 (12%)			28 (9%)		
NLR			** < 0.001**			0.838			** < 0.001**			** < 0.001**
≤ median	299 (39%)	31.5 (23.2–39.8)		49 (48%)	102.2 (70.2–134.2)		151 (42%)	39.8 (27.8–51.8)		103 (34%)	11.5 (9.0–14.0)	
> median	299 (39%)	14.5 (11.9–17.1)		45 (45%)	79.2 (0–165.9)		148 (41%)	19.6 (14.6–24.6)		102 (33%)	8.3 (7.2–9.4)	
Missing	171 (22%)			7 (7%)			62 (17%)			102 (33%)		
LLR			** < 0.001**			0.236			** < 0.001**			** < 0.001**
≤ median	297 (39%)	31.5 (23.1–39.9)		47 (47%)	110.8 (87.9–133.7)		149 (41%)	36.9 (22.4–51.4)		102 (33%)	11.5 (9.0–14.0)	
> median	297 (39%)	13.9 (11.5–16.3)		47 (47%)	70.8 (43.6–98.0)		148 (41%)	20.4 (15.9–24.9)		101 (33%)	8.3 (7.0–9.6)	
Missing	175 (22%)			7 (6%)			64 (18%)			104 (34%)		
PLR			** < 0.001**			0.068			** < 0.001**			**0.021**
≤ median	284 (37%)	26.1 (18.1–34.1)		44 (44%)	110.8 (74.5–147.1)		139 (38%)	38.6 (20.9–56.3)		102 (33%)	11.5 (9.3–13.7)	
> median	283 (37%)	13.8 (11.4–16.2)		44 (44%)	79.2 (23.2–135.2)		139 (38%)	19.0 (14.5–23.5)		99 (32%)	8.2 (7.1–9.3)	
Missing	202 (26%)			13 (12%)			83 (23%)			106 (35%)		
SIRI			** < 0.001**			0.738			**0.001**			**0.020**
≤ median	299 (39%)	27.4 (20.5–34.3)		47 (47%)	109.3 (86.3–132.3)		151 (42%)	37.1 (23.6–50.6)		102 (33%)	10.8 (8.9–12.7)	
> median	298 (39%)	14.5 (12.2–16.8)		47 (47%)	79.2 (20.6–137.8)		148 (41%)	22.0 (16.6–27.4)		102 (33%)	8.7 (7.2–10.2)	
Missing	172 (22%)			7 (6%)			62 (17%)			103 (34%)		

Bold values indicate statistical significance

### Association of systemic inflammation, stages and weight loss

Higher scores of mGPS were statistically significantly associated with higher levels of NLR (*p* < 0.001), LLR (*p* < 0.001), PLR (*p* < 0.001) and SIRI (*p* < 0.001).

Higher systemic inflammation parameters were statistically significantly associated with more advanced stages. In particular, patients with advanced or metastatic settings had statistically significantly higher NLR (median: 2.94 versus 3.2 versus 3.87; *p* < 0.001), LLR (median: 4.44 versus 4.67 versus 5.57; *p* < 0.001), PLR (median: 160.72 versus 170.77 versus 200.00; p < 0.001) and SIRI (median: 1.5 versus 1.6 versus 2.5; *p* < 0.001) compared to localized or locally advanced stages (see Table 1). Higher mGPS values were also associated with higher tumour stages (*p* < 0.001) (see Table 2 and Supplementary Fig. 4).

Furthermore, there was an association between tumour location and systemic inflammatory biomarkers (see Supplementary Figs. 4 and 5). PLR (*p* = 0.014), mGPS (*p* = 0.007) and SIRI (*p* = 0.011) were statistically significantly associated with tumour location, whereas NLR (*p* = 0.113) and LLR (*p* = 0.081) were not.

Weight loss at initial diagnosis was also statistically associated with higher systemic inflammation parameters (NLR: median 3.1 versus 3.6; *p* = 0.006; LLR: 4.6 versus 5.1; *p* = 0.009; PLR: 160.0 versus 194.7; *p* < 0.001; SIRI: 1.6 versus 2.1; *p* = 0.015; mGPS: *p* = 0.002; weight loss no versus yes, respectively).

Concerning BMI, NLR (*p* = 0.352), LLR (*p* = 0.545), SIRI (*p* = 0.540) and mGPS (*p* = 0.756) had no statistically significant association, but higher PLR levels were associated with lower BMI levels (median: 198.42 versus 184.73 versus 167.50; *p* = 0.031). Associations of systemic inflammation, stages, tumour location, weight loss and BMI are visualised in Supplementary Figs. 1–5.

More severe disease stages were associated with higher rates of weight loss at initial diagnosis (39% versus 51% versus 61%; *p* < 0.001), but not with BMI (*p* = 0.108). However, lower BMI levels were statistically significantly associated with weight loss at first diagnosis (*p* < 0.001).

Furthermore, alcohol abuse was statistically significantly associated with higher NLR (*p* = 0.015), LLR (*p* = 0.012) and SIRI (*p* = 0.016) levels, but not with PLR (*p* = 0.351) and mGPS (*p* = 0.338). Nicotine abuse was statistically significantly associated with PLR (*p* < 0.001), but not with NLR (*p* = 0.055), LLR (*p* = 0.070), SIRI (*p* = 0.721) and mGPS (*p* = 0.052).

*Helicobacter pylori* infection was associated with lower SIRI levels (*p* = 0.044), but not with other inflammatory parameters (NLR: *p* = 0.507; LLR: *p* = 0.434; PLR: *p* = 0.524; mGPS: *p* = 0.406).

### Systemic inflammation and overall survival

**Fig. 1:**
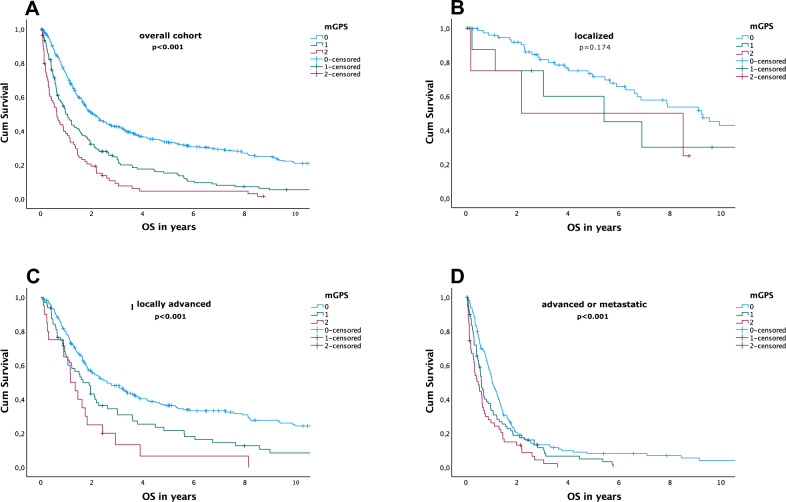
Kaplan–Meier overall survival (OS) estimates for modified Glasgow Prognostic Score (mGPS) in the overall cohort (**A**), localised setting (**B**), locally advanced setting (**C**) and advanced or metastatic setting (**D**)

Higher mGPS scores were statistically significantly associated with shorter OS in the overall cohort (24.9 versus 11.9 versus 7.6 months; HR 1.74, 95% CI 1.549–1.056; *p* < 0.001) as well as in locally advanced (31.1 versus 19.8 versus 13.9 months, HR 1.561, 95% CI 1.274–1.912; *p* < 0.001) and in advanced or metastatic settings (12.3 versus 7.3 versus 5.8 months; HR 1.377, 95% CI 1.777–1.611; *p* < 0.001). These results are visualised in Fig. 1 and are also elaborated in Table 2.

In addition, NLR, LLR, PLR and SIRI were grouped into high level (> median) and low level (≤ median) cohorts based on the medians of the respective subgroups (see Table 1). There was a statistically significant association between higher levels and OS in the overall as well as locally advanced and advanced or metastatic subgroups (see Tables 1 and 2). Kaplan–Meier survival curves are shown in Supplementary Figs. 6–9.

### Multivariate analyses

In the overall cohort, stage setting (HR 1.654, 95% CI 1.404–1.949; *p* < 0.001), weight loss (HR 1.64, 95% CI 1.153–2.333, *p* = 0.006), Lauren classification (HR 1.466, 95% CI 1.144–1.88; *p* = 0.003) and treatment (HR 0.197, 95% CI 0.084–0.462; *p* < 0.001) were still associated with the OS in a multivariate analysis. Other variables are listed in Supplementary Table 4.

In the locally advanced subgroup, only weight loss (HR 1.58, 95% CI 1.136–2.197; *p* = 0.007), treatment (HR 0.083, 95% CI 0.019–0.361; *p* < 0.001) and mGPS (HR 1.397, 95% CI 1.068–1.828; *p* = 0.015) were statistically significant in the multivariate analysis. Other parameters are shown in Supplementary Table 5.

Concerning the advanced and metastatic subgroup, none of the values stayed statistically significant in a multivariate analysis (age: *p* = 0.208; Lauren classification: *p* = 0.213; Her2: *p* = 0.398; PD-L1 CPS: *p* = 0.676; NLR: *p* = 0.260; LLR: linearly dependent covariate; PLR: *p* = 0.811; SIRI: *p* = 0.617; mGPS: *p* = 0.128; treatment: linearly dependent covariate).

For resectable setting, no multivariate analysis was performed as no variables had statistically significant association with OS in univariate analyses.

## Discussion

Inflammatory parameters play a key role in cancer development and progression. Yet, prognostic effects of systemic inflammatory parameters have rarely been studied in large Western cohorts with gastroesophageal adenocarcinoma and it is unclear which inflammatory parameter is the best predictor for overall survival. The results of our analyses shed some light on this issue, as they show that mGPS is a feasible prognostic tool to estimate overall survival in a large, representable European cohort of gastroesophageal adenocarcinoma patients.

Although the leucocyte ratios NLR, LLR and PLR also showed prognostic potential in univariate analyses, only high mGPS scores at first diagnosis could be identified as an independent tool in multivariate analysis of the locally advanced cohort. NLR and LLR are surmised to reflect an increased inflammatory response to tumour cells (Buonacera et al. 2022). Whether this mechanism is associated with immune cell infiltration in tumour tissue is widely discussed (Choi et al. 2016; Puhr et al. 2022), but no clear association has been proven yet. In addition, PLR may represent the important role of platelet-derived tumour angiogenesis maintained by vascular endothelial growth factor and platelet-derived growth factor leading to tumour progression and dissemination. Thus, several trials have shown distinctive associations of these ratios with tumour progression and outcome in gastroesophageal cancer patients as well as patients with other cancer entities (Namikawa et al. 2016; Starzer et al. 2021; Pikuła et al. 2022; Miyamoto et al. 2018; Lian et al. 2015; Kim et al. 2022; Petrillo et al. 2018). Yet, the definition of clear cut-off levels for risk stratification is still lacking (Schiefer et al. 2022). This might be due to the fact that although higher levels are associated with more severe tumour stages and survival, differences between ratios are small and effected by outliers (see Supplementary Figs. 1–3). Systemic inflammation parameters not only serve as prognostic tools but could also be used to predict response to immunotherapy. Initial emerging data from translational research programmes of clinical trials indicate potential usability of these markers, particularly NLR, in decision-making between chemotherapy and immunotherapy doublet (Chau et al. 2022). Although the implementation of these ratios outside of academic evaluations is still not feasible, further translational research of clinical trials will provide valuable data and an improved understanding of these markers for the clinical routine.

The mGPS, which is based on CRP and albumin levels, presents a more robust and feasible tool than systemic inflammatory ratios as it is easily calculated and only encompasses three levels (score 0 to 2). mGPS has been shown to be associated with postoperative outcome in gastroesophageal cancer patients in several Asian cohorts (Jiang et al. 2012; Chen et al. 2020; Hirashima et al. 2014; Zhang et al. 2022a, b). Yet, data on Western cohorts and comparisons between tumour stages are scarce.

In our cohort, mGPS was also associated with more severe tumour staging. Further analyses of stage subgroups showed that mGPS was an independent prognostic marker in multivariate analysis of the locally advanced cohort. These results suggest that although scores are the highest in advanced and metastatic settings, the prognostic value is the greatest in locally advanced settings. This finding is in line with perioperative studies of Asian cohorts (Jiang et al. 2012; Chen et al. 2020; Hirashima et al. 2014; Zhang et al. 2022a, b). Recently published data from a Romanian cohort, investigating leucocyte ratios, also suggests that the prognostic value of inflammatory biomarkers is the greatest in locally advanced settings (Nechita et al. 2022). This is further supported by two European studies investigating inflammatory biomarkers in resectable gastric cancer (Pikuła et al. 2022; Schietroma et al. 2022). However, multivariate analyses and recommendations for clear cut-off levels of leucocyte ratios are lacking in these trials. Furthermore, the role of tumour location in systemic inflammatory processes is unclear.

Thus, based on our findings, mGPS might pose a feasible prognostic tool in everyday practice and could improve patient management in perioperative settings by raising physicians’ awareness of early recurrences as well as perioperative morbidity in patients with higher scores. Yet, the exact mechanisms behind these consistent results of associations between elevated systemic inflammatory biomarkers and inferior outcome in locally advanced settings have not been identified and need to be further explored.

In a recently published analysis of advanced gastroesophageal cancer patients undergoing first-line treatment from the phase III EXPAND trial, mGPS could be associated with sarcopenia (Hacker et al. 2022). Our results show that elevated systemic inflammation parameters were associated with weight loss rather than with BMI. Although not directly assessed in the current cohort, sarcopenia, which is often characterised by weight loss independent of BMI, might also be a large contributor to higher mGPS scores in our cohort. However, concerning multivariate analyses in the overall cohort, mGPS stayed statistically significantly associated with the OS and so did weight loss. Thus, the prognostic value of mGPS might not only be associated with sarcopenia but other values. This theory is supported by the findings of Hacker et al. as mGPS carried full prognostic information for OS, but direct causal path from sarcopenia to survival was lacking (Hacker et al. 2022).

Moreover, elevated systemic inflammatory parameters and consequently higher mGPS levels might be caused by circulating tumour cells in more advanced stages, as these tumours are known to be associated with higher levels of circulating tumour components. Circulating tumour DNA (ctDNA) has gained scientific interest as a key prognostic value after tumour resection in various cancers (Moati et al. 2021). First attempts to guide adjuvant therapy by the analysis of ctDNA in colorectal cancer have shown promising results (Tie et al. 2022). Prognostic effects of ctDNA in gastroesophageal cancer patients have recently been published and feasibility as a widely used prognostic markers is under evaluation (van Velzen et al. 2022; Shoda et al. 2017; Iqbal et al. 2019). Moreover, trials investigating further systemic treatment in patients with high ctDNA levels in gastroesophageal cancer are underway and might shed some light on the association of ctDNA with systemic inflammatory parameters such as mGPS.

Furthermore, it was hypothesised that systemic inflammatory biomarkers might pose as predictive parameters for response to immunotherapy. A recently published retrospective analysis of hepatocellular carcinoma patients undergoing checkpoint inhibition suggests that NLR and PLR are independent negative prognostic factors (Muhammed et al. 2021). Similar associations could be drawn in other tumour entities and with various immunotherapeutic agents (Stares et al. 2022; Ye et al. 2023). Yet, so far, only tumour tissue-based markers, such as PD-L1, tumour mutational burden (TMB-H) and mismatch repair deficiency/microsatellite instability (dMMR/MSI-H), are approved biomarkers for administration of immunotherapy (Food and Drug Administration 2017a, b, 2020, 2021). First attempts to correlate these markers with soluble counterparts form liquid biopsies showed promising potential (Mair et al. 2020; Shin et al. 2023). In addition, a post hoc examination of clinical data from the CheckMate-648 trial suggested a potential association between baseline NLR and different response rates in esophageal squamous cell carcinoma patients receiving immunotherapy, chemotherapy or immuno-chemotherapy (Chau et al. 2022). However, data on gastroesophageal adenocarcinoma are still scarce as immunotherapy has only been approved within the last few years. This is underlined by the relatively small subgroup of patients who were tested for PD-L1 or dMMR/MSI-H expression in our cohort.

Furthermore, patients without PD-L1, TMB-H or dMMR/MSI-H tumours in some cases also show exceedingly promising responses leading to the hypothesis that other biomarkers might provide more sufficient predictive value (Zhang et al. 2021). Thus, further research is warranted to investigate the predictive potential of systemic inflammatory biomarkers in respect to immunotherapeutic strategies.

Due to the retrospective design of our analyses, further exploration of associations with immuno-histochemical staining, such as PD-L1 and dMMR/MSI-H, as well as underlying infectious or autoimmune diseases, therapeutic strategies and longitudinal evaluation of systemic inflammatory parameters could not be performed and therefore pose limitations to our work. In addition, the large timespan of patient recruitment poses a potential bias and is included in our analyses. However, year of first diagnosis was only significantly associated with the OS in the overall but not in the stage-based subgroups as throughout the last decades, more severe cases have been transferred and treated at our department. Despite these limitations of the analysis, our results pose an important cornerstone for further evaluation of mGPS in gastroesophageal adenocarcinoma patients.

In conclusion, higher systemic inflammatory biomarkers were statistically significantly associated with more severe tumour stages and weight loss at first diagnosis. Concerning subgroups based on tumour stages, mGPS showed robust prognostic value in patients with locally advanced disease, which is in line with results from Asian cohorts. Further analyses are warranted to explore the cause and potential clinical applications of elevated mGPS scores.

### Supplementary Information

Below is the link to the electronic supplementary material.

Supplementary Fig. 1: Boxplots of neutrophil-to-lymphocyte ratios (NLR), lymphocyte-to-leucocyte ratios (LLR), platelet-to-lymphocyte ratios (PLR) and systemic inflammation response index (SIRI) according to tumour stage setting (**A**–**D**). *p*-values estimated with Kruskal–Wallis method (PDF 74 KB)

Supplementary Fig. 2: Boxplots of neutrophil-to-lymphocyte ratios (NLR), lymphocyte-to-leucocyte ratios (LLR), platelet-to-lymphocyte ratios (PLR) and systemic inflammation response index (SIRI) according to weight loss (**A**–**D**). *p*-values estimated with Kruskal–Wallis method (PDF 52 KB)

Supplementary Fig. 3: Boxplots of neutrophil-to-lymphocyte ratios (NLR), lymphocyte-to-leucocyte ratios (LLR), platelet-to-lymphocyte ratios (PLR) and systemic inflammation response index (SIRI) according to body mass index (BMI) (**A**–**D**). *p*-values estimated with Kruskal–Wallis method (PDF 64 KB)

Supplementary Fig. 4: Histograms of modified Glasgow prognostic score (mGPS) according to tumour stage setting (**A**), weight loss (**B**) and body mass index (BMI; Fig. 3). *P*-values estimated with chi-square-method (PDF 80 KB)

Supplementary Fig. 5: Boxplots of neutrophil-to-lymphocyte ratios (NLR), lymphocyte-to-leucocyte ratios (LLR), platelet-to-lymphocyte ratios (PLR) and systemic inflammation response index (SIRI) according to tumour location (**A**–**D**). *p*-values estimated with Kruskal–Wallis method (PDF 55 KB)

Supplementary Fig. 6: Kaplan–Meier survival estimates of neutrophil-to-lymphocyte ratios (NLR) in association with the overall survival (OS) in a cohort of 769 patients with gastroesophageal adenocarcinoma (**A**) and sub-cohort of localised (**B**), locally advanced (**C**) and advanced or metastatic cancer patients (**D**). *p*-values estimated with log-rank test (PDF 153 KB)

Supplementary Fig. 7: Kaplan–Meier survival estimates of lymphocyte-to-leucocyte ratios (LLR) in association with the overall survival (OS) in a cohort of 769 patients with gastroesophageal adenocarcinoma (**A**) and sub-cohort of localised (**B**), locally advanced (**C**) and advanced or metastatic cancer patients (**D**). *p*-values estimated with log-rank test (PDF 153 KB)

Supplementary Fig. 8: Kaplan–Meier survival estimates of platelet-to-lymphocyte ratios (PLR) in association with the overall survival (OS) in a cohort of 769 patients with gastroesophageal adenocarcinoma (**A**) and subcohort of localised (**B**), locally advanced (**C**) and advanced or metastatic cancer patients (**D**). *p*-values estimated with log-rank test (PDF 154 KB)

Supplementary Fig. 9: Kaplan–Meier survival estimates of systemic inflammation response index (SIRI) in association with the overall survival (OS) in a cohort of 769 patients with gastroesophageal adenocarcinoma (**A**) and sub-cohorts of localised (**B**), locally advanced (**C**) and advanced or metastatic cancer patients (**D**). *p*-values estimated with log-rank test (PDF 155 KB)

Supplementary file10 (DOCX 18 KB)

Supplementary file11 (DOCX 18 KB)

Supplementary file12 (DOCX 16 KB)

Supplementary file13 (DOCX 16 KB)

Supplementary file14 (DOCX 15 KB)

## Data Availability

The data that support the findings of this study are available from the corresponding author upon reasonable request.

## Abbreviations

NLR: Neutrophil-to-lymphocyte ratio
LLR: Leucocyte-to-lymphocyte ratio
PLR: Platelet-to-lymphocyte ratio
SIRI: Systemic inflammation response index
mGPS: Modified Glasgow Prognostic Score
OS: Overall survival
CRP: C-reactive protein
HR: Hazard ratio
CI: Confidential interval
SD: Standard deviation
HER2: Human epidermal growth factor receptor 2
WBC: White blood cells
SPSS: Statistical package for the social sciences
BMI: Body mass index
ctDNA: Circulating tumour DNA

## Declarations

### Additional information

This study was performed within the PhD thesis of Hannah Christina Puhr with the title “Immunological, genetic and prognostic factors related to outcome in patients with upper gastrointestinal tract cancer” in the Doctoral Program N790 “Clinical & Experimental Oncology” at the Medical University of Vienna. Parts of this analysis were presented as a poster at the World Congress of Gastrointestinal Cancer 2021 (June 30–July 3, 2021) and the first author HC.P. was awarded a “young investigator award” for the work.

## References

[CR1] Bender R, Lange S (2001). Adjusting for multiple testing–when and how?. J Clin Epidemiol.

[CR2] Buonacera A, Stancanelli B, Colaci M, Malatino L (2022). Neutrophil to lymphocyte ratio: an emerging marker of the relationships between the immune system and diseases. Int J Mol Sci.

[CR3] Chau I, Ajani J, Doki Y, Xu J, Wyrwicz L, Motoyama S, Ogata T, Kawakami H, Hsu C, Adenis A, El Hajbi F, Di Bartolomeo M, Braghiroli M, Holtved E, Blum Murphy M, Abdullaev S, Soleymani S, Lei M, Kato K, Kitagawa Y (2022). O-3 Nivolumab (NIVO) plus chemotherapy (chemo) or ipilimumab (IPI) vs chemo as first-line treatment for advanced esophageal squamous cell carcinoma (ESCC): expanded efficacy and safety analyses from CheckMate 648. Ann Oncol.

[CR4] Chen Y-R, Chen Y-L, Ouyang S-S, Hui-Wen Xu, Li P, He L-J, Zhu S-L (2020). Prognostic efficacy of preoperative mGPS, SIS and LCS in patients with gastric cancer. Clin Chim Acta.

[CR5] Choi Y, Kim JW, Nam KH, Kim JW, Ahn SH, Park DJ, Lee KW, Lee HS, Kim HH (2016). Systemic inflammation is associated with the density of immune cells in the tumor microenvironment of gastric cancer. Ann Oncol.

[CR6] Dhakras P, Uboha N, Horner V, Reinig E, Matkowskyj KA (2020). Gastrointestinal cancers: current biomarkers in esophageal and gastric adenocarcinoma. Transl Gastroenterol Hepatol.

[CR7] Food and Drug Administration (2017a) FDA grants accelerated approval to pembrolizumab for advanced gastric cancer. Accessed 30/06/2021. https://www.fda.gov/drugs/resources-information-approved-drugs/fda-grants-accelerated-approval-pembrolizumab-advanced-gastric-cancer.

[CR8] Food and Drug Administration (2017b) FDA grants accelerated approval to pembrolizumab for first tissue/site agnostic indication. Accessed 30/06/2021. https://www.fda.gov/drugs/resources-information-approved-drugs/fda-grants-accelerated-approval-pembrolizumab-first-tissuesite-agnostic-indication.

[CR9] Food and Drug Administration (2020) FDA approves pembrolizumab for adults and children with TMB-H solid tumors. Accessed 01/11/2020. https://www.fda.gov/drugs/drug-approvals-and-databases/fda-approves-pembrolizumab-adults-and-children-tmb-h-solid-tumors.

[CR10] Food and Drug Administration (2021) FDA approves nivolumab in combination with chemotherapy for metastatic gastric cancer and esophageal adenocarcinoma. Accessed 30/06/2021. https://www.fda.gov/drugs/resources-information-approved-drugs/fda-approves-nivolumab-combination-chemotherapy-metastatic-gastric-cancer-and-esophageal

[CR11] Hacker UT, Hasenclever D, Baber R, Linder N, Busse H, Obermannova R, Zdrazilova-Dubska L, Valik D, Lordick F (2022). Modified Glasgow prognostic score (mGPS) is correlated with sarcopenia and dominates the prognostic role of baseline body composition parameters in advanced gastric and esophagogastric junction cancer patients undergoing first-line treatment from the phase III EXPAND trial. Ann Oncol.

[CR12] Hirashima K, Watanabe M, Shigaki H, Imamura Y, Ida S, Iwatsuki M, Ishimoto T, Iwagami S, Baba Y, Baba H (2014). Prognostic significance of the modified Glasgow prognostic score in elderly patients with gastric cancer. J Gastroenterol.

[CR13] Huang C, Li Z, Zhang Z, Xia X, Xu D, Zhao A, Zhao G (2020). Prognostic value and association of systemic inflammation for patients with stage IV gastric cancer. Acta Gastroenterol Belg.

[CR14] Iqbal M, Roberts A, Starr J, Mody K, Kasi PM (2019). Feasibility and clinical value of circulating tumor DNA testing in patients with gastric adenocarcinomas. J Gastrointest Oncol.

[CR15] Jiang X, Hiki N, Nunobe S, Kumagai K, Kubota T, Aikou S, Sano T, Yamaguchi T (2012). Prognostic importance of the inflammation-based Glasgow prognostic score in patients with gastric cancer. Br J Cancer.

[CR16] Jomrich G, Paireder M, Kristo I, Baierl A, Ilhan-Mutlu A, Preusser M, Asari R, Schoppmann SF (2021). High systemic immune-inflammation index is an adverse prognostic factor for patients with gastroesophageal adenocarcinoma. Ann Surg.

[CR17] Kim M-R, Sol Kim A, Choi H-I, Jung J-H, Park JY, Ko H-J (2020). Inflammatory markers for predicting overall survival in gastric cancer patients: a systematic review and meta-analysis. PLoS One.

[CR18] Kim SG, Eom BW, Yoon H, Kim YW, Ryu KW (2022). Prognostic value of preoperative systemic inflammatory parameters in advanced gastric cancer. J Clin Med.

[CR19] Laird BJ, Kaasa S, McMillan DC, Fallon MT, Hjermstad MJ, Fayers P, Klepstad P (2013). Prognostic factors in patients with advanced cancer: a comparison of clinicopathological factors and the development of an inflammation-based prognostic system. Clin Cancer Res.

[CR20] Li Z, Sun Y, Ye F, Ma D, Xianli Yin Wu, Zhuang XY, Qin S, Zhang Y, Kangsheng Gu, Zhao K, Xiao J, Cheng Y, Bai Y, Luo S, Wang L, Wang C, Cui Yi, Mei L, Shen L (2021). First-line pembrolizumab plus chemotherapy versus chemotherapy in patients with advanced esophageal cancer: Chinese subgroup analysis of KEYNOTE-590. J Clin Oncol.

[CR21] Lian L, Xia YY, Zhou C, Shen XM, Li XL, Han SG, Zheng Y, Mao ZQ, Gong FR, Wu MY, Chen K, Tao M, Li W (2015). Application of platelet/lymphocyte and neutrophil/lymphocyte ratios in early diagnosis and prognostic prediction in patients with resectable gastric cancer. Cancer Biomark.

[CR22] Liu Z, Ge H, Miao Z, Shao S, Shi H, Dong C (2021). Dynamic changes in the systemic inflammation response index predict the outcome of resectable gastric cancer patients. Front Oncol.

[CR23] Lordick F, Carneiro F, Cascinu S, Fleitas T, Haustermans K, Piessen G, Vogel A, Smyth EC (2022). Gastric cancer: ESMO Clinical Practice Guideline for diagnosis, treatment and follow-up. Ann Oncol.

[CR24] Mair MJ, Pajenda S, Ilhan-Mutlu A, Steindl A, Kiesel B, Widhalm G, Dieckmann K, Feldmann K, Hainfellner J, Marosi C, Müllauer L, Wagner L, Preusser M, Berghoff AS (2020). Soluble PD-L1 is associated with local and systemic inflammation markers in primary and secondary brain tumours. ESMO Open.

[CR25] Matsumoto T, Ohki S, Kaneta A, Matsuishi A, Maruyama Y, Yamada L, Tada T, Hanayama H, Watanabe Y, Hayase S, Okayama H, Sakamoto W, Momma T, Saze Z, Kono K (2023). Systemic inflammation score as a preoperative prognostic factor for patients with pT2–T4 resectable gastric cancer: a retrospective study. BMC Surg.

[CR26] Miyamoto R, Inagawa S, Sano N, Tadano S, Adachi S, Yamamoto M (2018). The neutrophil-to-lymphocyte ratio (NLR) predicts short-term and long-term outcomes in gastric cancer patients. Eur J Surg Oncol.

[CR27] Moati E, Taly V, Garinet S, Didelot A, Taieb J, Laurent-Puig P, Zaanan A (2021). Role of circulating tumor DNA in gastrointestinal cancers: current knowledge and perspectives. Cancers (basel).

[CR28] Moehler MH, Shitara K, Garrido M, Salman P, Shen L, Wyrwicz L, Yamaguchi K, Skoczylas T, Bragagnoli ASC, Liu T, Schenker M, Yanez PE, Tehfe M, Li M, Cullen D, Memaj A, Lei M, Xiao H, Janjigian YY, Ajani JA (2021). First-line (1L) nivolumab (NIVO) plus chemotherapy (chemo) versus chemo in advanced gastric cancer/gastroesophageal junction cancer/esophageal adenocarcinoma (GC/GEJC/EAC): expanded efficacy and safety data from CheckMate 649. J Clin Oncol.

[CR29] Muhammed A, Fulgenzi CAM, Dharmapuri S, Pinter M, Balcar L, Scheiner B, Marron TU, Jun T, Saeed A, Hildebrand H, Muzaffar M, Navaid M, Naqash AR, Gampa A, Ozbek U, Lin JY, Perone Y, Vincenzi B, Silletta M, Pillai A, Wang Y, Khan U, Huang YH, Bettinger D, Abugabal YI, Kaseb A, Pressiani T, Personeni N, Rimassa L, Nishida N, Di Tommaso L, Kudo M, Vogel A, Mauri FA, Cortellini A, Sharma R, D'Alessio A, Ang C, Pinato DJ (2021). The systemic inflammatory response identifies patients with adverse clinical outcome from immunotherapy in hepatocellular Carcinoma. Cancers (basel).

[CR30] Namikawa T, Munekage E, Munekage M, Maeda H, Yatabe T, Kitagawa H, Kobayashi M, Hanazaki K (2016). Evaluation of systemic inflammatory response biomarkers in patients receiving chemotherapy for unresectable and recurrent advanced gastric cancer. Oncology.

[CR31] Nechita VI, Al-Hajjar N, Moiş E, Furcea L, Nechita MA, Graur F (2022). Inflammatory ratios as predictors for tumor invasiveness, metastasis, resectability and early postoperative evolution in gastric cancer. Curr Oncol.

[CR32] Obermannová R, Alsina M, Cervantes A, Leong T, Lordick F, Nilsson M, van Grieken NCT, Vogel A, Smyth EC (2022). Oesophageal cancer: ESMO Clinical Practice Guideline for diagnosis, treatment and follow-up. Ann Oncol.

[CR33] Petrillo A, Laterza MM, Tirino G, Pompella L, Ventriglia J, Pappalardo A, Famiglietti V, Martinelli E, Ciardiello F, Orditura M, Galizia G, De Vita F (2018). Systemic-inflammation-based score can predict prognosis in metastatic gastric cancer patients before first-line chemotherapy. Future Oncol.

[CR34] Pikuła A, Skórzewska M, Pelc Z, Mlak R, Gęca K, Sędłak K, Ciseł B, Kwietniewska M, Rawicz-Pruszyński K, Polkowski WP (2022). Prognostic value of systemic inflammatory response markers in patients undergoing neoadjuvant chemotherapy and gastrectomy for advanced gastric cancer in the Eastern European Population. Cancers (basel).

[CR35] Proctor MJ, Morrison DS, Talwar D, Balmer SM, O'Reilly DS, Foulis AK, Horgan PG, McMillan DC (2011). An inflammation-based prognostic score (mGPS) predicts cancer survival independent of tumour site: a Glasgow Inflammation Outcome Study. Br J Cancer.

[CR36] Puhr H, Hatziioannou T, Beer A, Kain R, Jomrich G, Paireder M, Schoppmann S, Berghoff A, Preusser M, Ilhan-Mutlu A (2022). P-175 Local inflammatory biomarkers and their association with systemic inflammation as well as overall survival in primary metastatic gastroesophageal cancer patients. Ann Oncol.

[CR37] Rha SY, Ku GY, Kim HS, Chung HC, Amlashi FG, Maru DM, Fein CA, Tang LH, Zhou W, Wu T, Peter SA, Kelsen DP, Ajani JA (2022). PD-L1 expression and overall survival in Asian and western patients with gastric cancer. Future Oncol.

[CR38] Schiefer S, Wirsik NM, Kalkum E, Seide SE, Nienhüser H, Müller B, Billeter A, Büchler MW, Schmidt T, Probst P (2022). Systematic review of prognostic role of blood cell ratios in patients with gastric cancer undergoing surgery. Diagnostics (basel).

[CR39] Schietroma M, Romano L, Schiavi D, Pessia B, Mattei A, Fiasca F, Carlei F, Giuliani A (2022). Systemic inflammation response index (SIRI) as predictor of anastomotic leakage after total gastrectomy for gastric cancer. Surg Oncol.

[CR40] Shin K, Kim J, Park SJ, Lee MA, Park JM, Choi MG, Kang D, Song KY, Lee HH, Seo HS, Lee SH, Kim B, Kim O, Park J, Kang N, Kim IH (2023). Prognostic value of soluble PD-L1 and exosomal PD-L1 in advanced gastric cancer patients receiving systemic chemotherapy. Sci Rep.

[CR41] Shitara K, Ajani JA, Moehler M, Garrido M, Gallardo C, Shen L, Yamaguchi K, Wyrwicz L, Skoczylas T, Bragagnoli AC, Liu T, Tehfe M, Elimova E, Bruges R, Zander T, de Azevedo S, Kowalyszyn R, Pazo-Cid R, Schenker M, Cleary JM, Yanez P, Feeney K, Karamouzis MV, Poulart V, Lei M, Xiao H, Kondo K, Li M, Janjigian YY (2022). Nivolumab plus chemotherapy or ipilimumab in gastro-oesophageal cancer. Nature.

[CR42] Shoda K, Ichikawa D, Fujita Y, Masuda K, Hiramoto H, Hamada J, Arita T, Konishi H, Komatsu S, Shiozaki A, Kakihara N, Okamoto K, Taniguchi H, Imoto I, Otsuji E (2017). Monitoring the HER2 copy number status in circulating tumor DNA by droplet digital PCR in patients with gastric cancer. Gastric Cancer.

[CR43] Stares M, Ding TE, Stratton C, Thomson F, Baxter M, Cagney H, Cumming K, Swan A, Ross F, Barrie C, Maclennan K, Campbell S, Evans T, Tufail A, Harrow S, Lord H, Laird B, MacKean M, Phillips I (2022). Biomarkers of systemic inflammation predict survival with first-line immune checkpoint inhibitors in non-small-cell lung cancer. ESMO Open.

[CR44] Starzer AM, Steindl A, Mair MJ, Deischinger C, Simonovska A, Widhalm G, Gatterbauer B, Dieckmann K, Heller G, Preusser M, Berghoff AS (2021). Systemic inflammation scores correlate with survival prognosis in patients with newly diagnosed brain metastases. Br J Cancer.

[CR45] Sung H, Ferlay J, Siegel RL, Laversanne M, Soerjomataram I, Jemal A, Bray F (2021). Global cancer statistics 2020: GLOBOCAN estimates of incidence and mortality worldwide for 36 cancers in 185 countries. CA Cancer J Clin.

[CR46] Tie J, Cohen JD, Lahouel K, Lo SN, Wang Y, Kosmider S, Wong R, Shapiro J, Lee M, Harris S, Khattak A, Burge M, Harris M, Lynam J, Nott L, Day F, Hayes T, McLachlan S-A, Lee B, Ptak J, Silliman N, Dobbyn L, Popoli M, Hruban R, Lennon AM, Papadopoulos N, Kinzler KW, Vogelstein B, Tomasetti C, Gibbs P (2022). Circulating tumor DNA analysis guiding adjuvant therapy in stage II colon cancer. N Engl J Med.

[CR47] van Velzen MJM, Creemers A, van den Ende T, Schokker S, Krausz S, Reinten RJ, Dijk F, van Noesel CJM, Halfwerk H, Meijer SL, Mearadji B, Derks S, Bijlsma MF, van Laarhoven HWM (2022). Circulating tumor DNA predicts outcome in metastatic gastroesophageal cancer. Gastric Cancer.

[CR48] Ye K, Xiao M, Li Z, He K, Wang J, Zhu L, Xiong W, Zhong Z, Tang Y (2023). Preoperative systemic inflammation response index is an independent prognostic marker for BCG immunotherapy in patients with non-muscle-invasive bladder cancer. Cancer Med.

[CR49] Zhang F, Zhang J, Zhao L, Zhai M, Zhang T, Yu D (2021). A PD-L1 negative advanced gastric cancer patient with a long response to PD-1 blockade after failure of systematic treatment: a case report. Front Immunol.

[CR50] Zhang S, Li JZ, Du T, Li HQ, Hu RH, Ma CY, Cui XM, Song C, Jiang XH (2022). The modified glasgow prognostic score predicts survival in gastric cancer patients with normal CEA and CA19-9. Can J Gastroenterol Hepatol.

[CR51] Zhang Z, Liu Z, Chen Z (2022). Comparison of treatment efficacy and survival outcomes between asian and western patients with unresectable gastric or gastro-esophageal adenocarcinoma: a systematic review and meta-analysis. Front Oncol.

