# Supplementary figures and images for: Systemic inflammatory biomarkers as prognostic tools in patients with gastroesophageal adenocarcinoma
Source: J Cancer Res Clin Oncol. 2023 Sep 26;149(19):17081–91. doi: 10.1007/s00432-023-05424-4 (PMC10657318; doi:10.1007/s00432-023-05424-4)

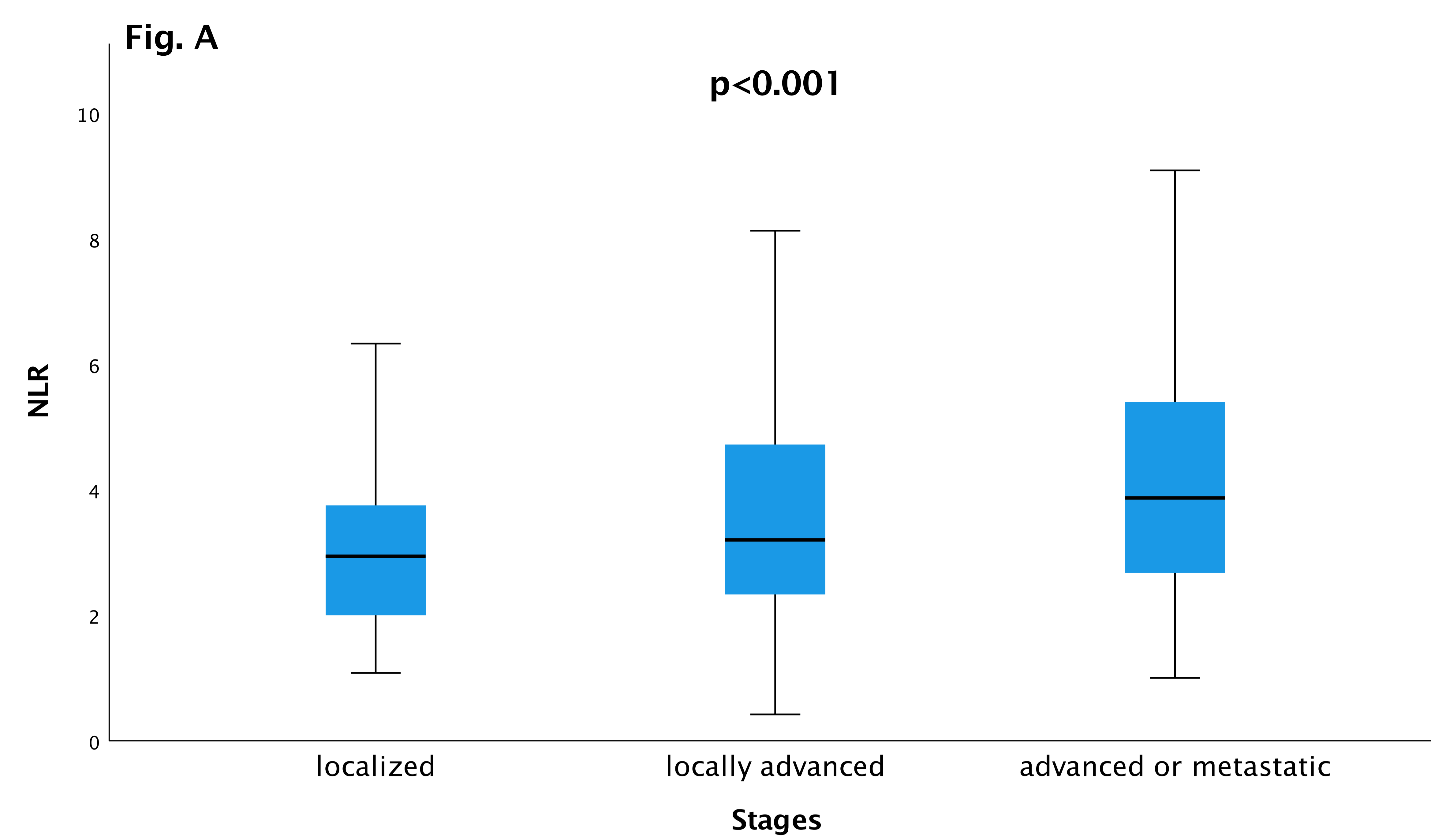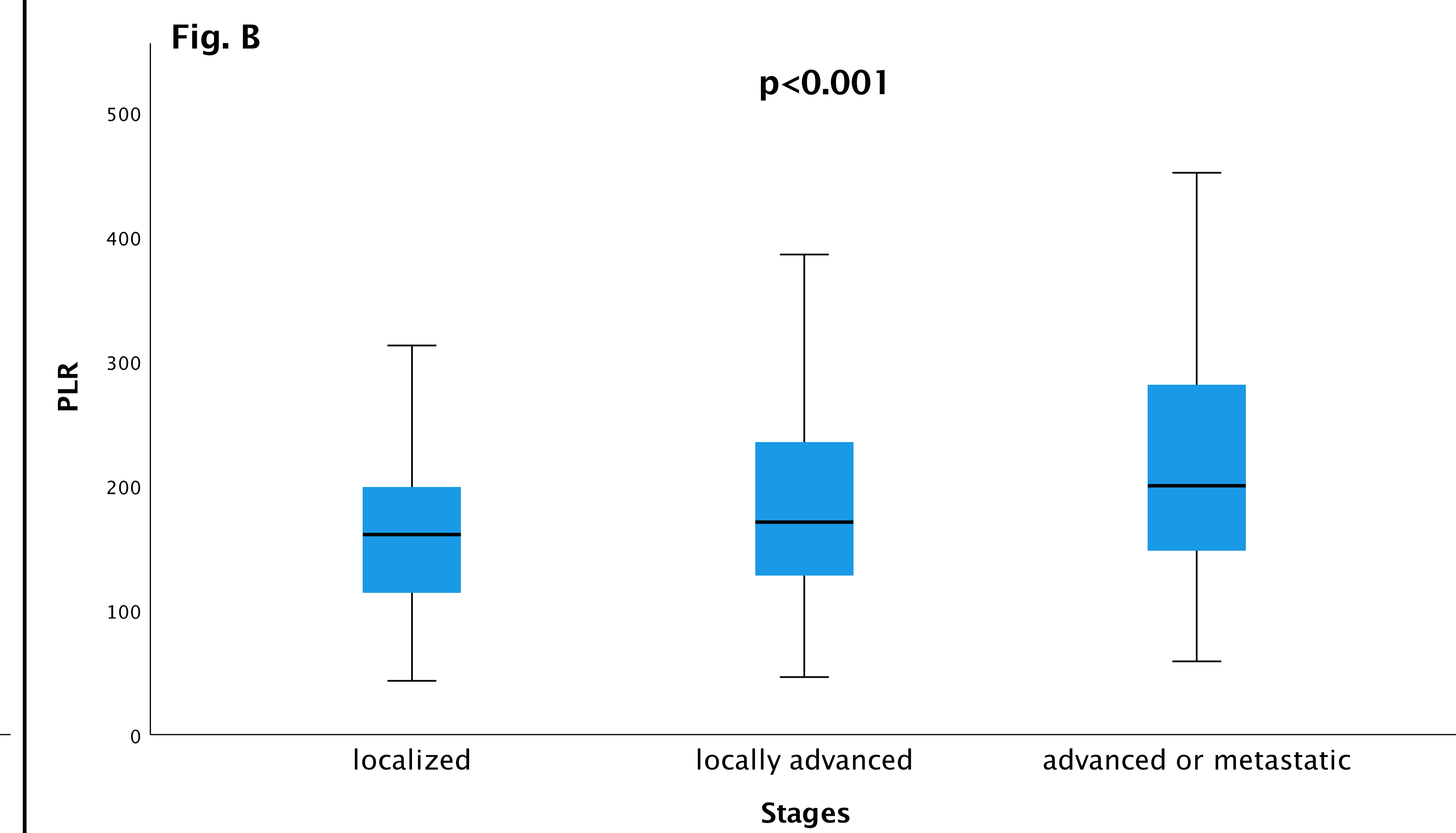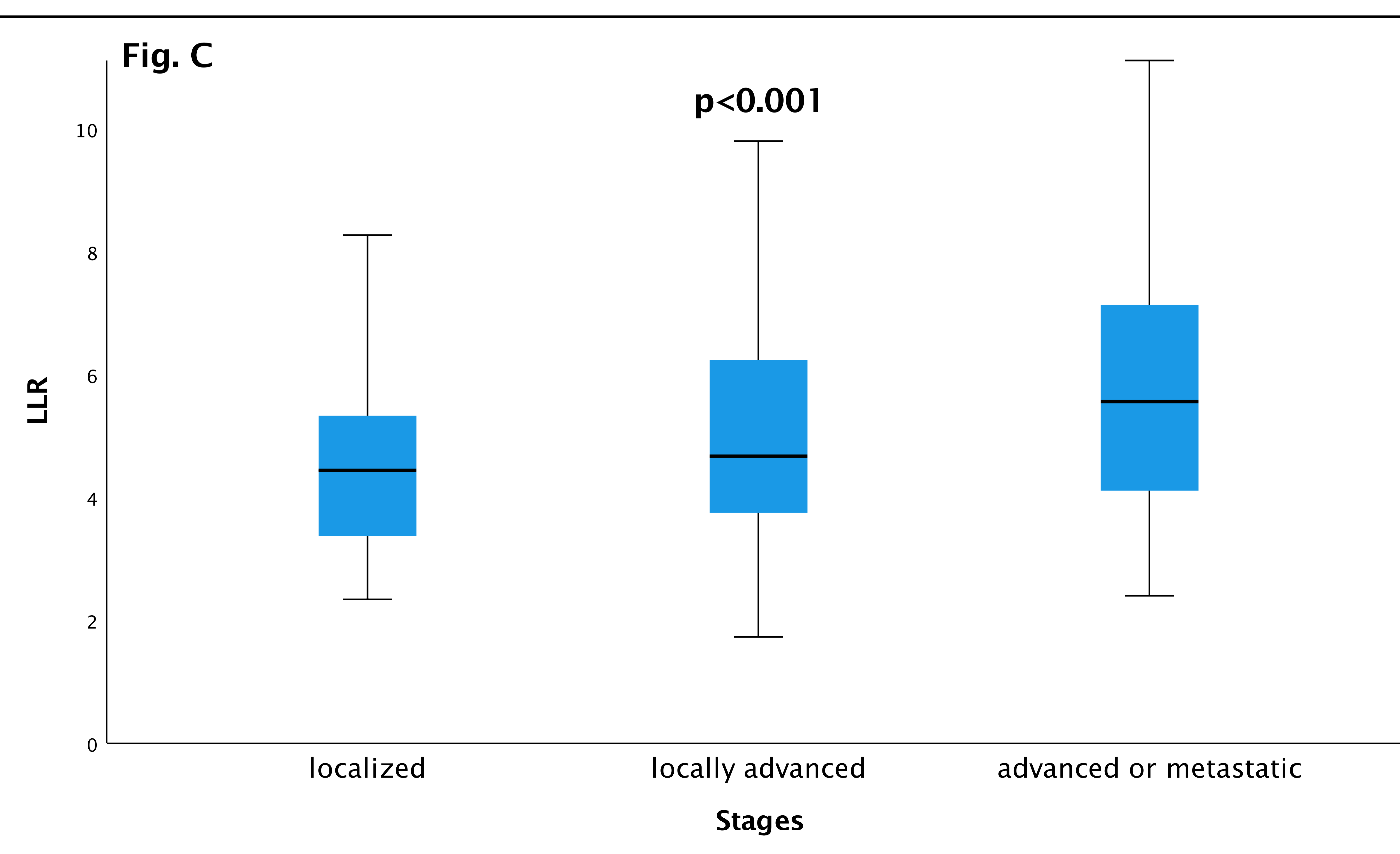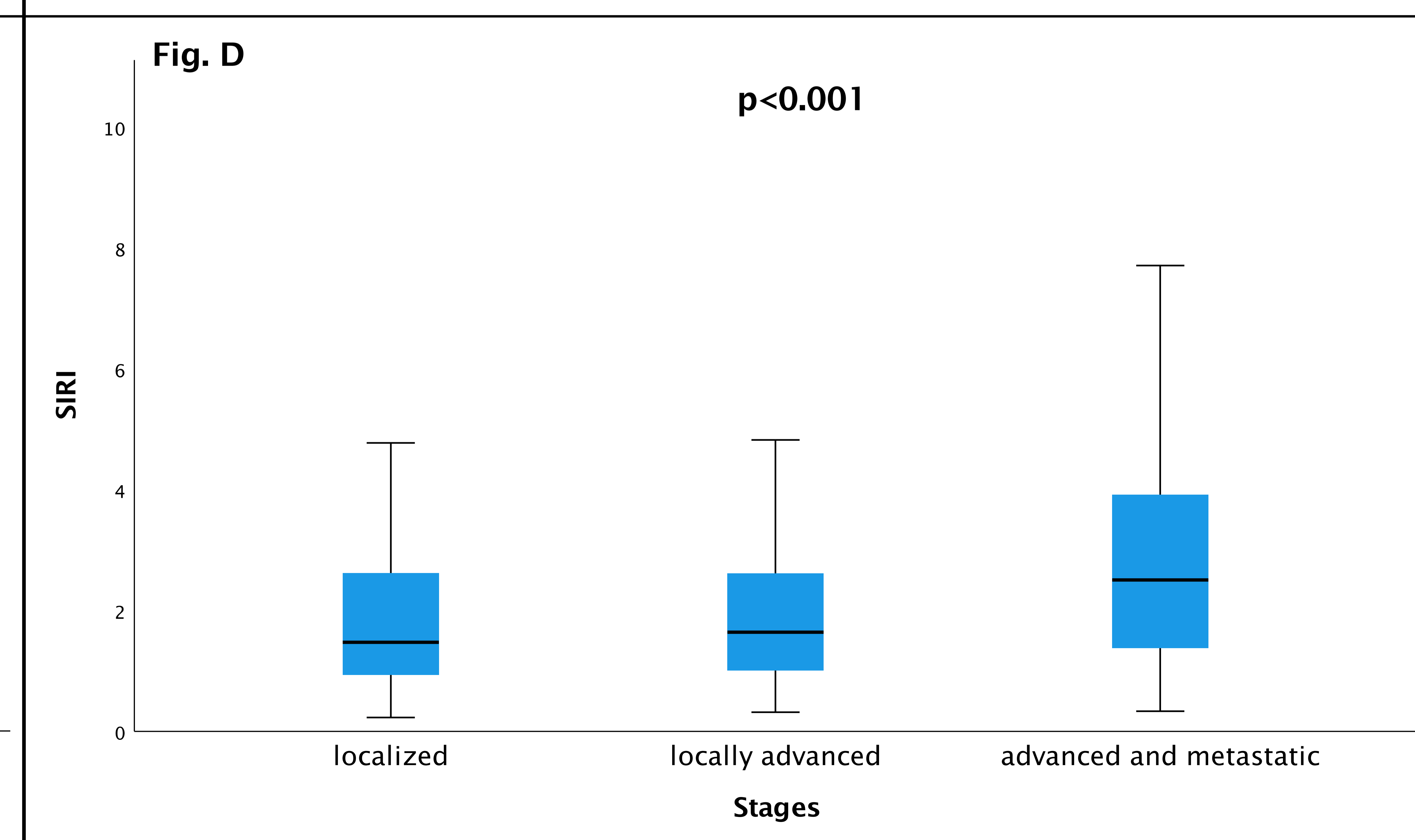

Supplement: Supplementary file 1 — Supplementary Fig. 1: Boxplots of neutrophil-to-lymphocyte ratios (NLR), lymphocyte-to-leucocyte ratios (LLR), platelet-to-lymphocyte ratios (PLR) and systemic inflammation response index (SIRI) according to tumour stage setting (A–D). p-values estimated with Kruskal–Wallis method (PDF 74 KB) [file 432_2023_5424_MOESM1_ESM.pdf]

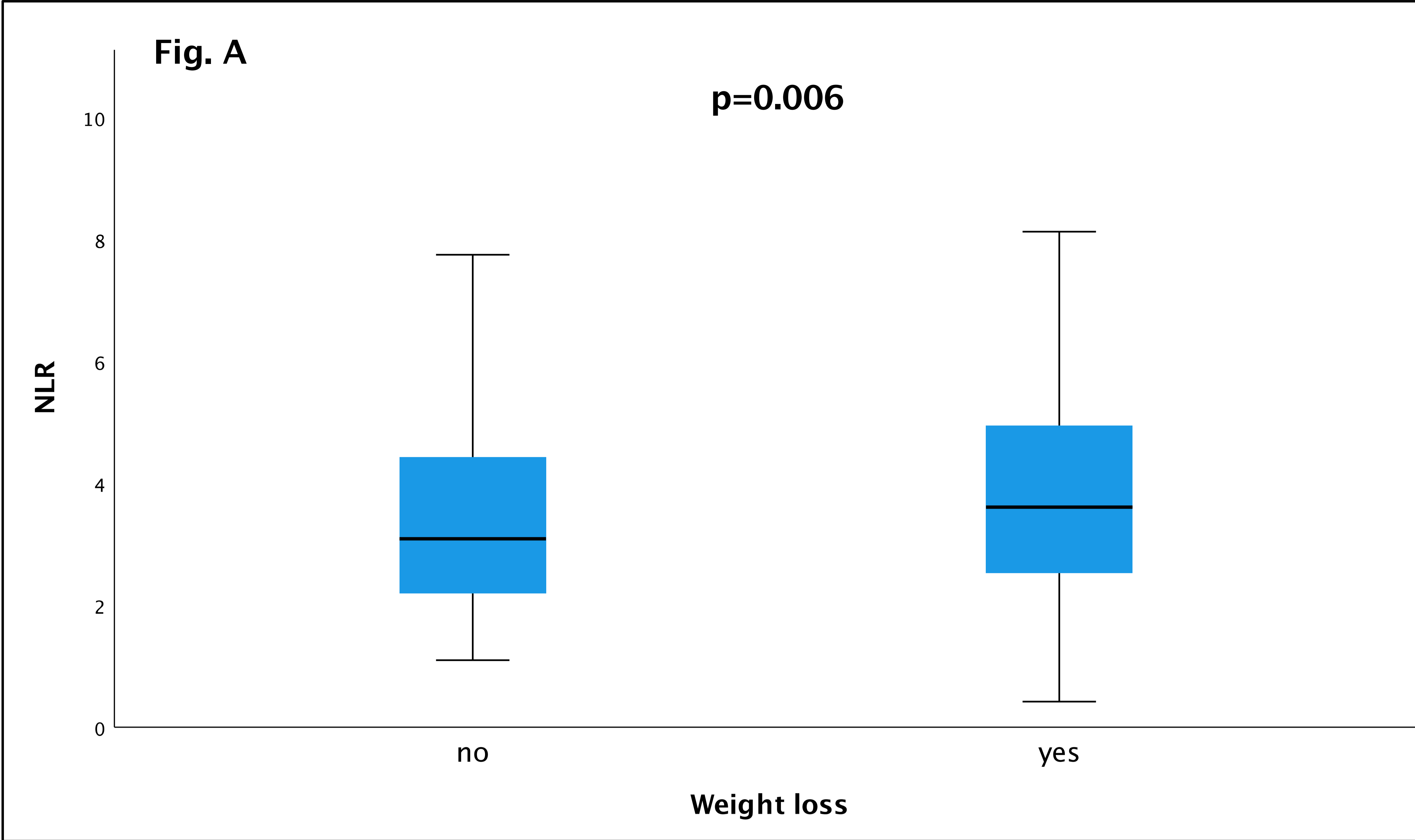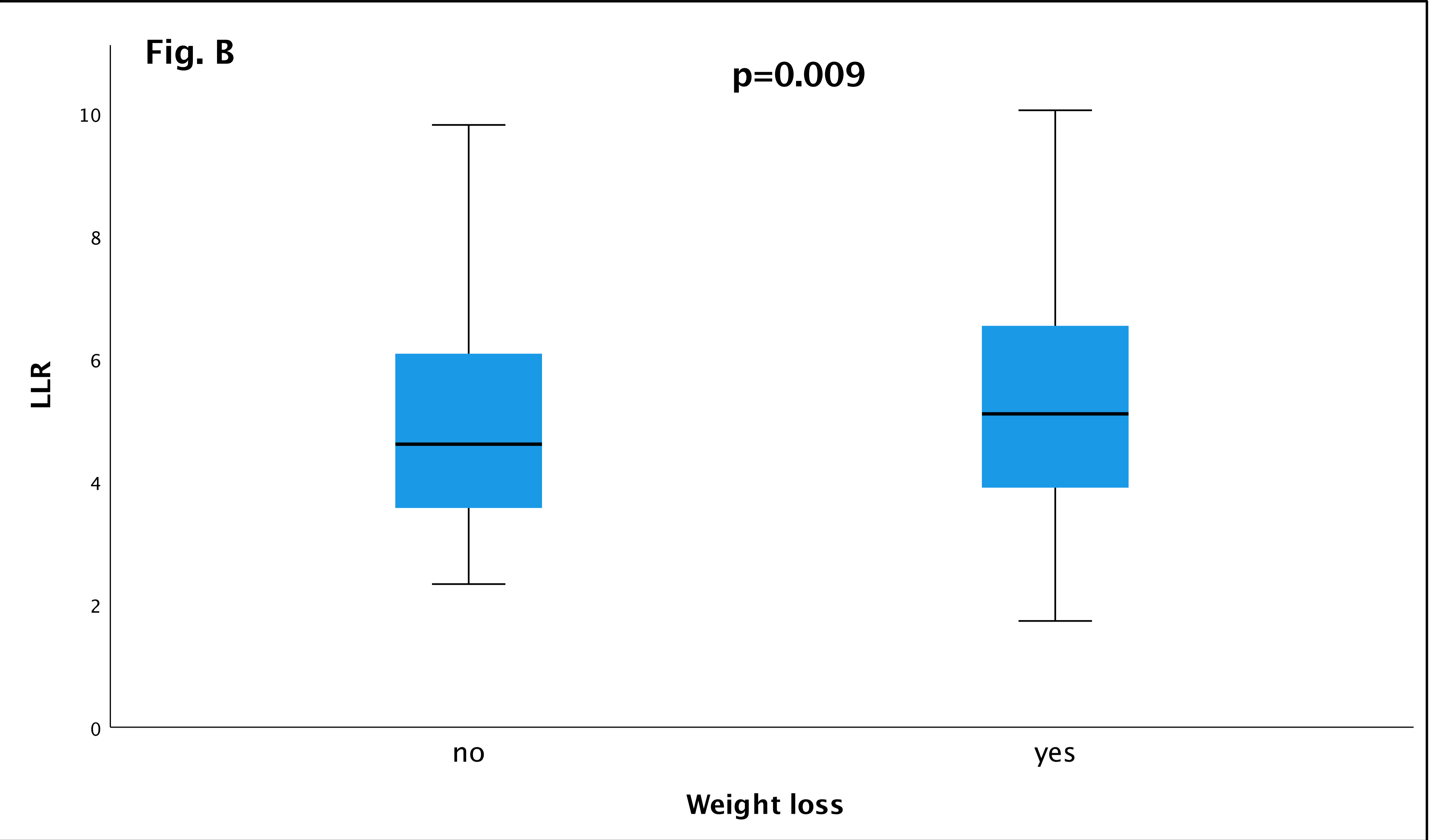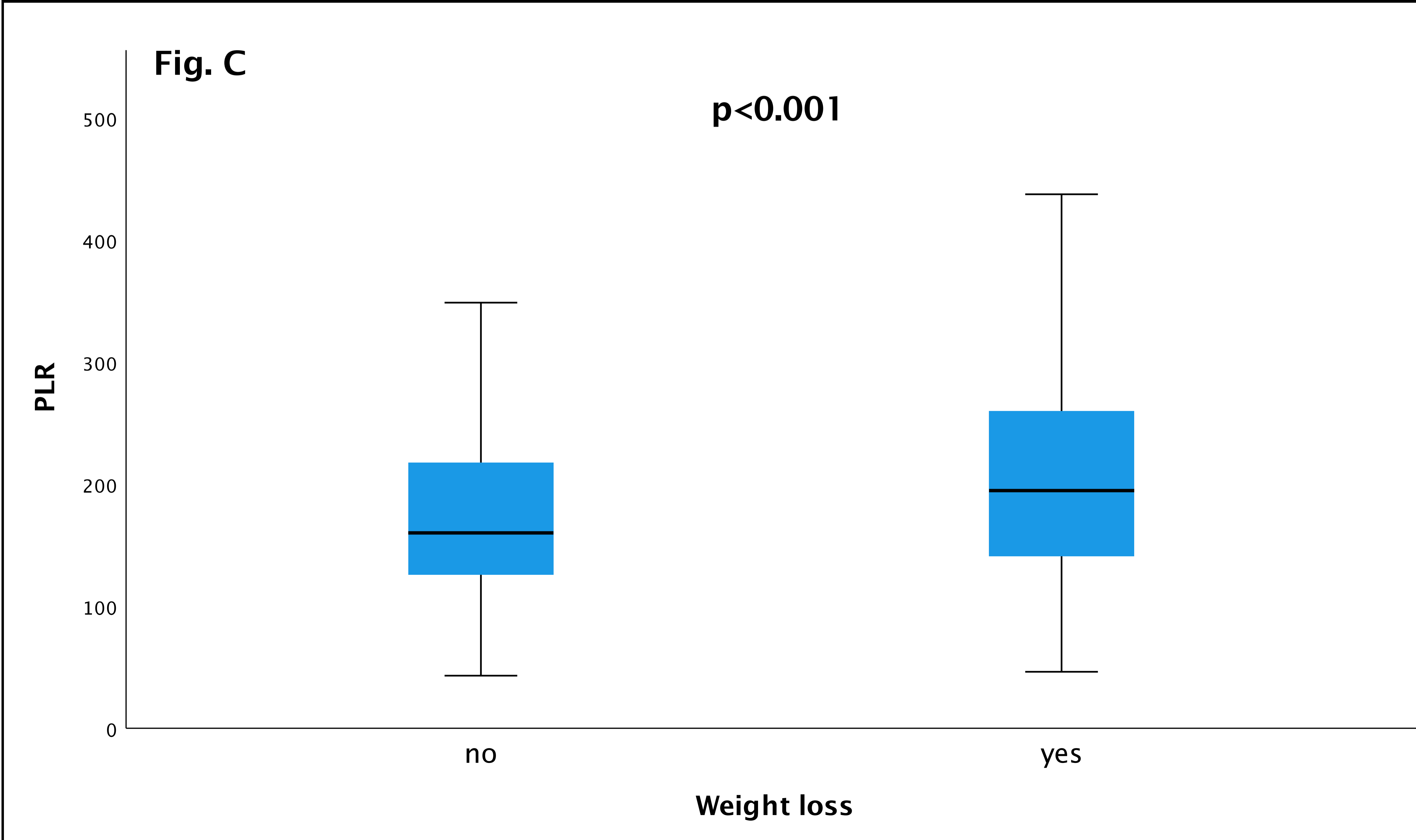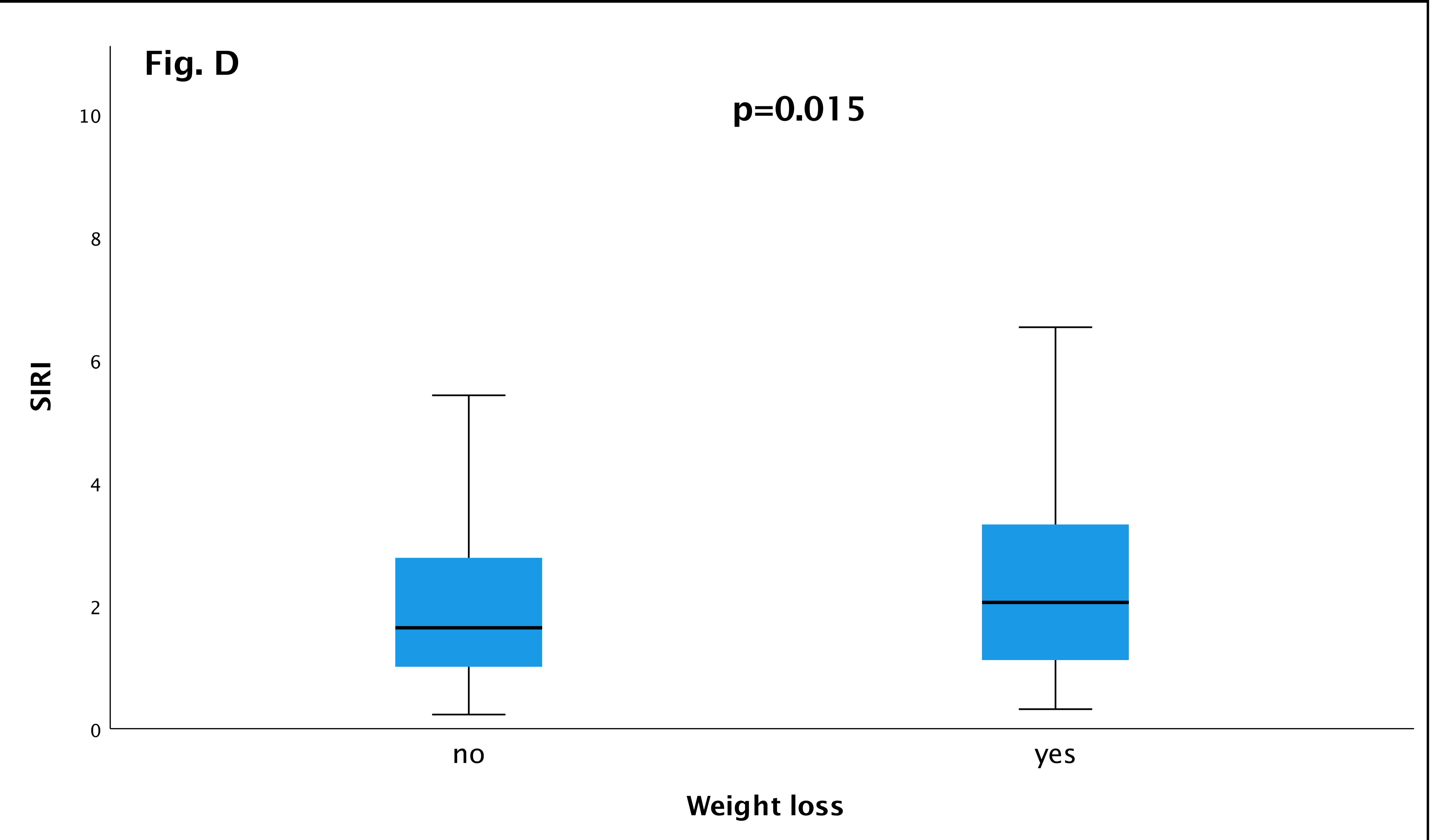

Supplement: Supplementary file 2 — Supplementary Fig. 2: Boxplots of neutrophil-to-lymphocyte ratios (NLR), lymphocyte-to-leucocyte ratios (LLR), platelet-to-lymphocyte ratios (PLR) and systemic inflammation response index (SIRI) according to weight loss (A–D). p-values estimated with Kruskal–Wallis method (PDF 52 KB) [file 432_2023_5424_MOESM2_ESM.pdf]

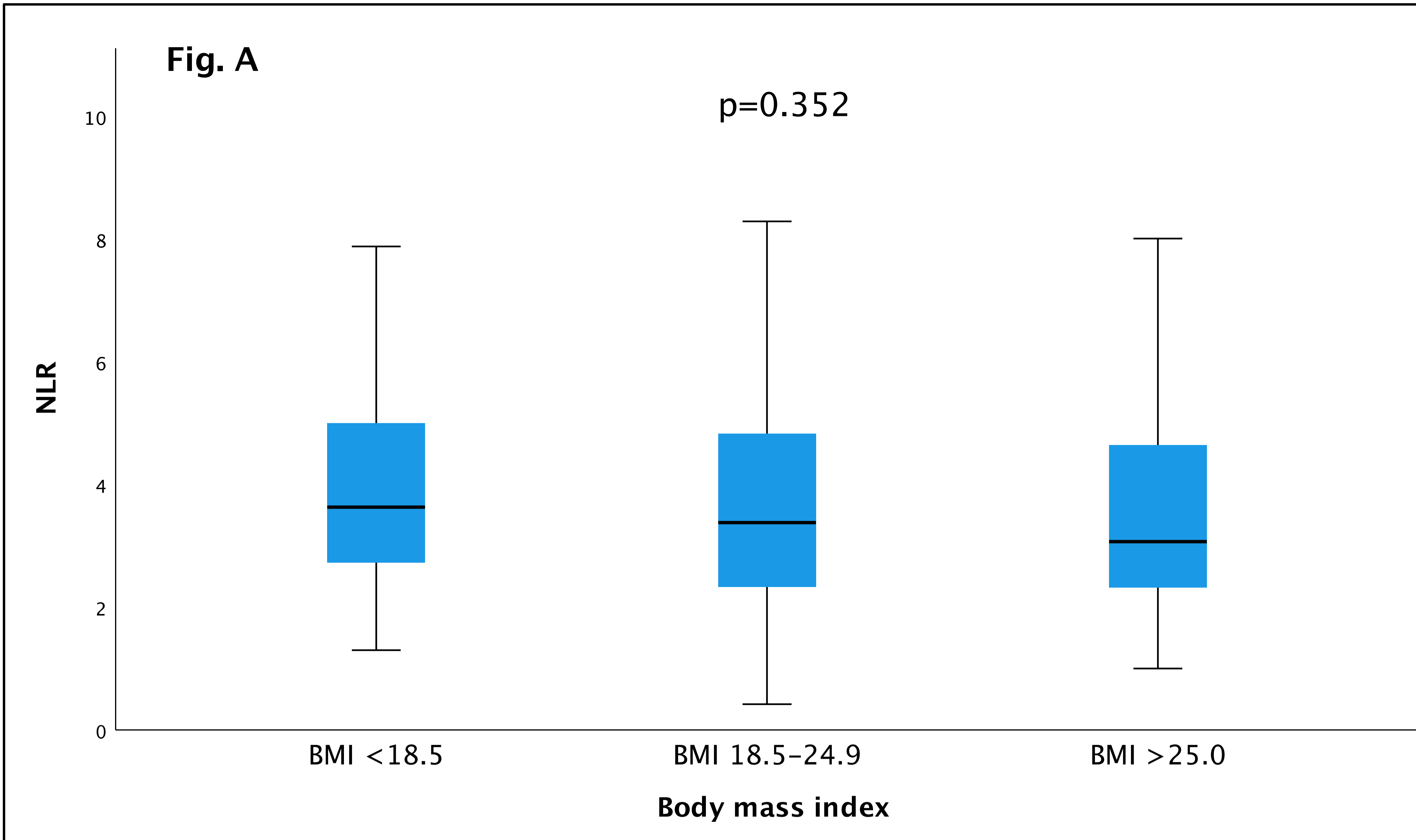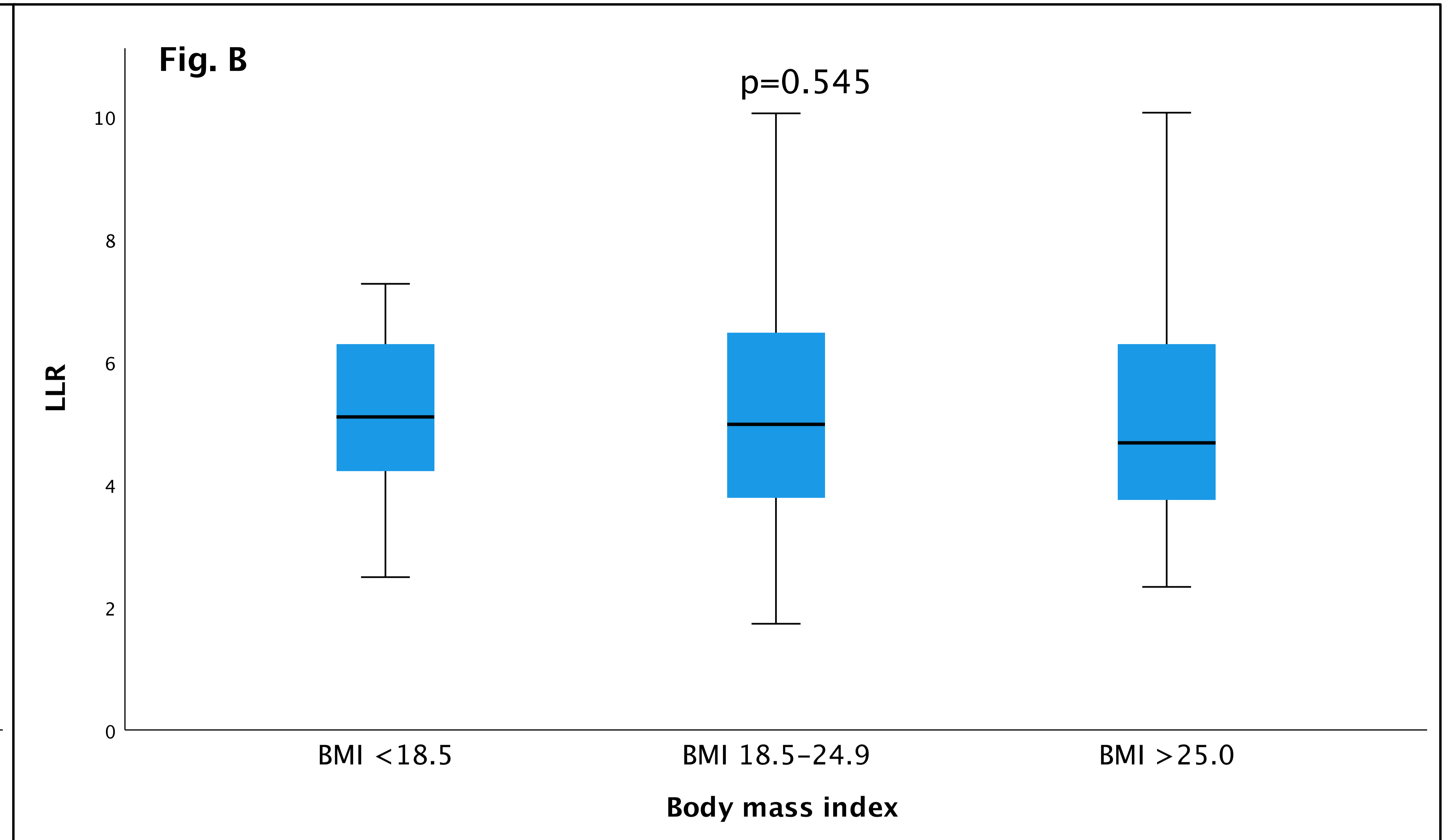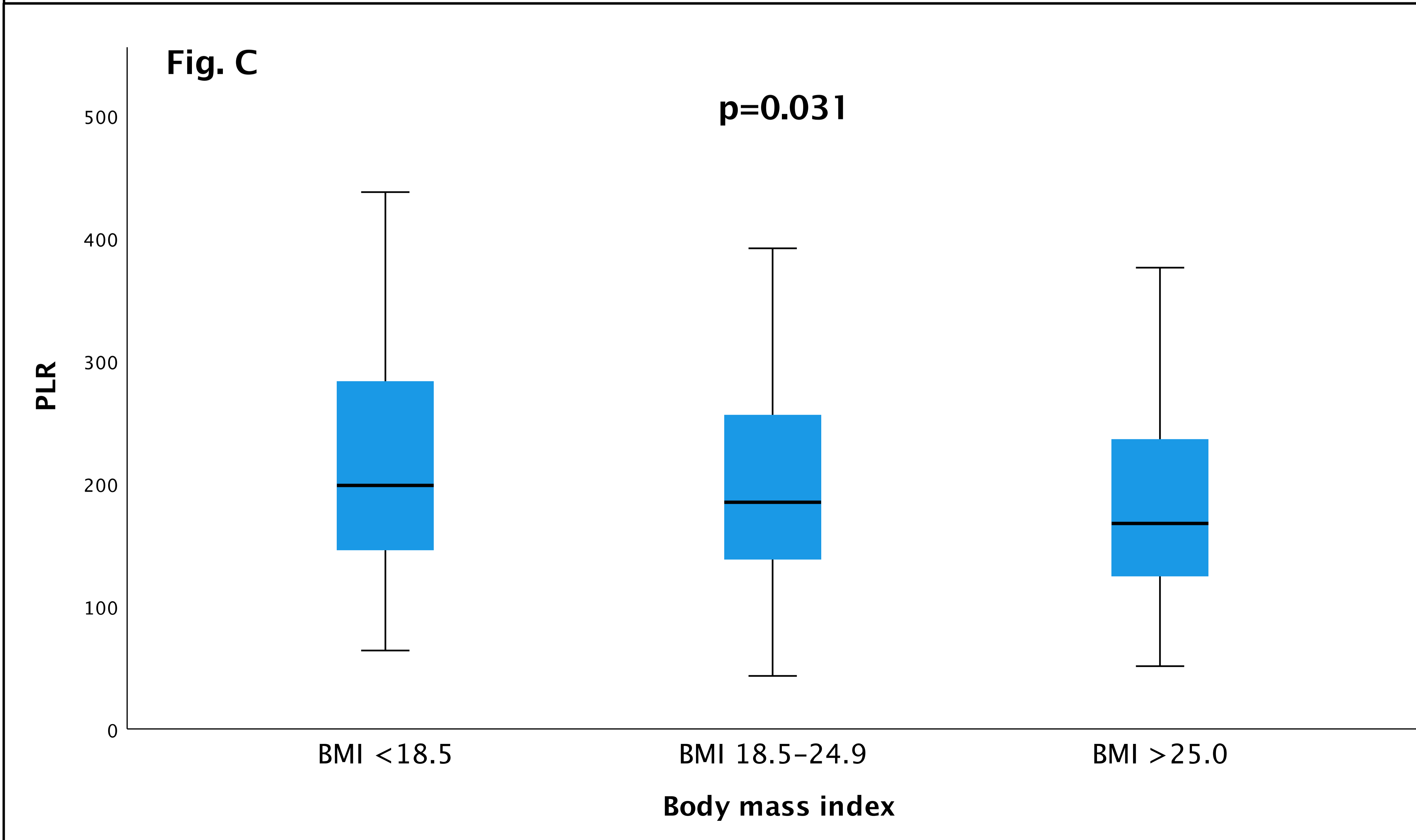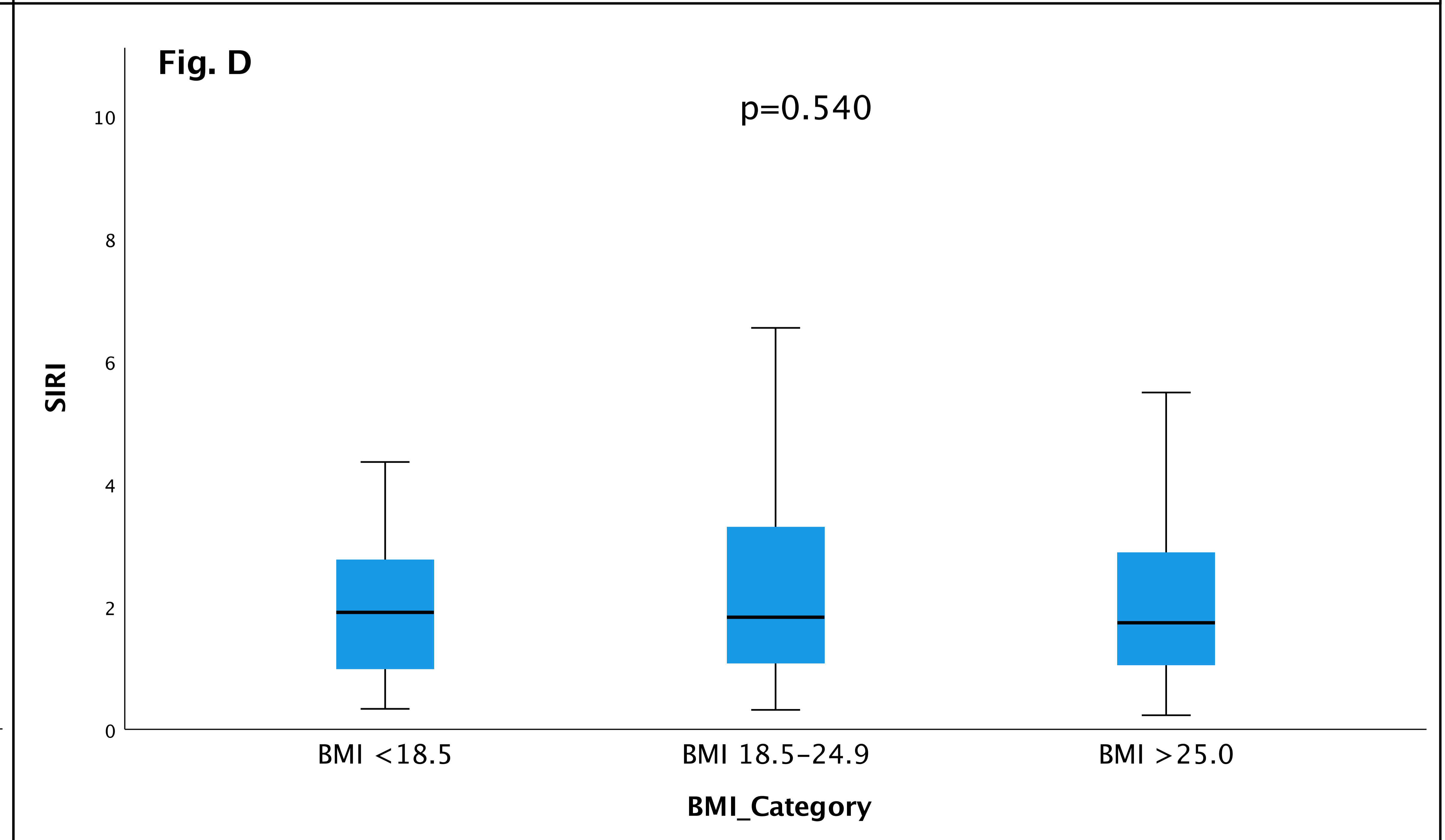

Supplement: Supplementary file 3 — Supplementary Fig. 3: Boxplots of neutrophil-to-lymphocyte ratios (NLR), lymphocyte-to-leucocyte ratios (LLR), platelet-to-lymphocyte ratios (PLR) and systemic inflammation response index (SIRI) according to body mass index (BMI) (A–D). p-values estimated with Kruskal–Wallis method (PDF 64 KB) [file 432_2023_5424_MOESM3_ESM.pdf]

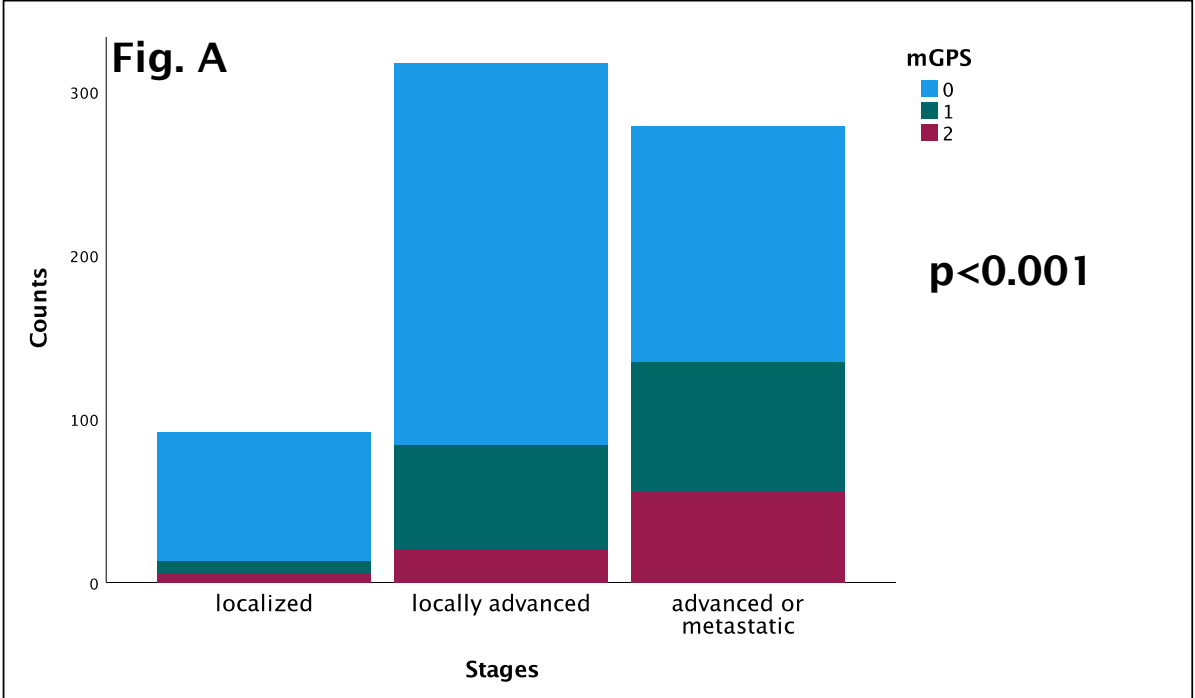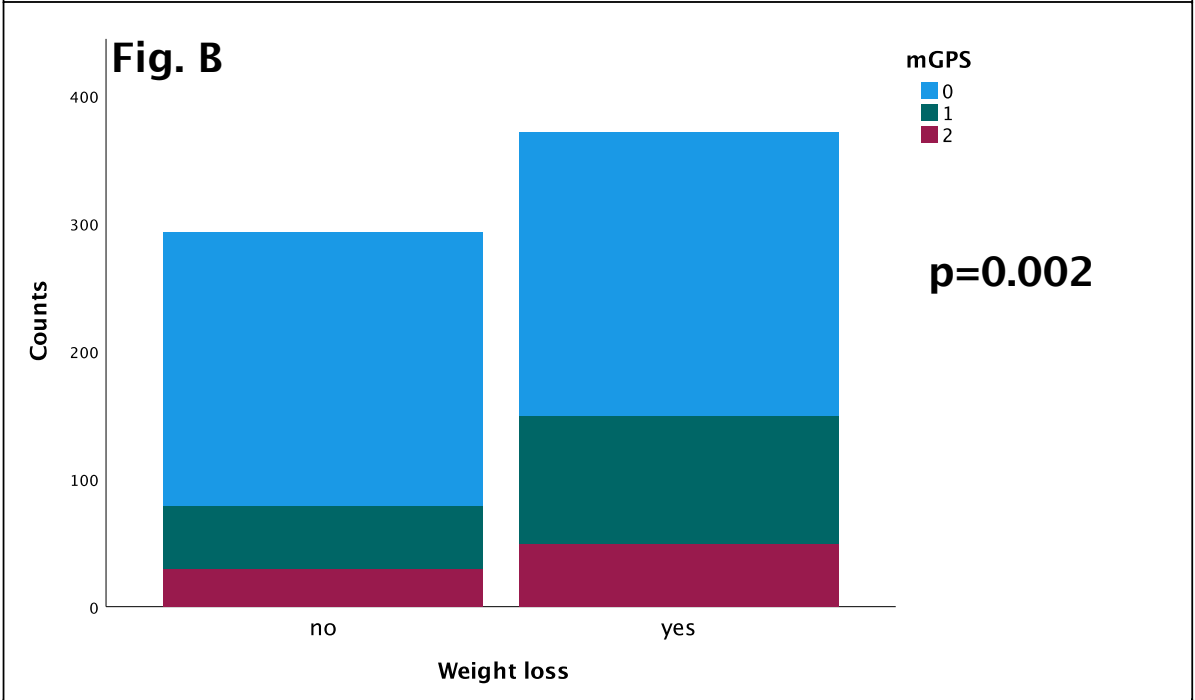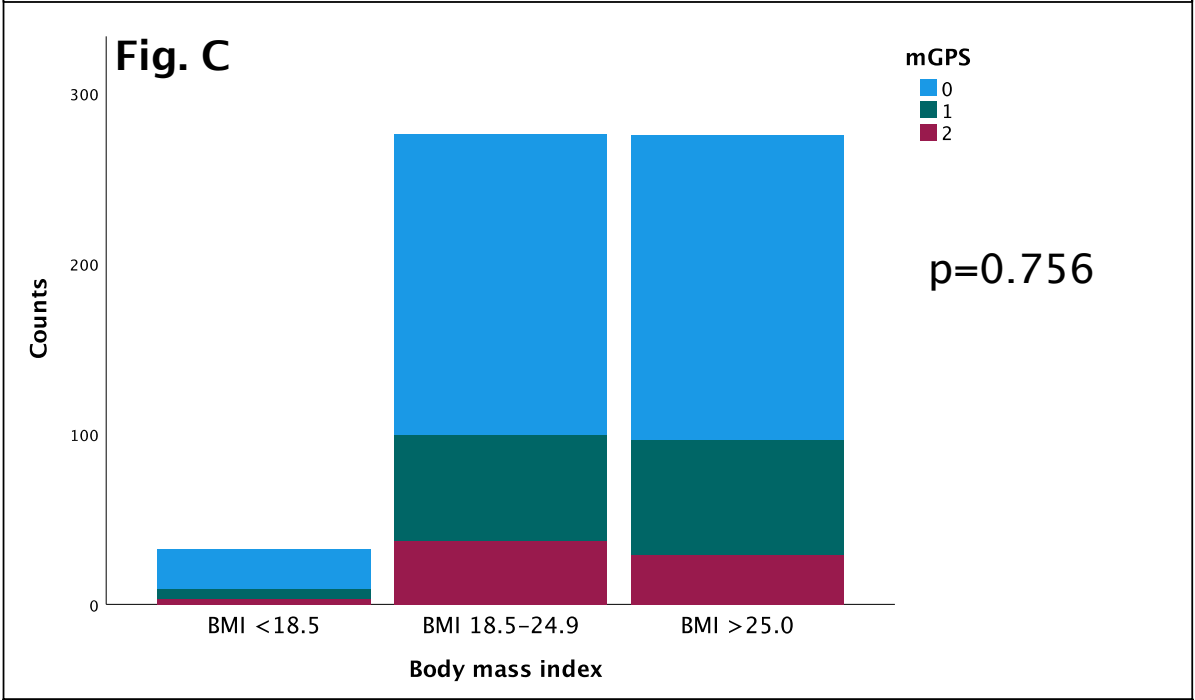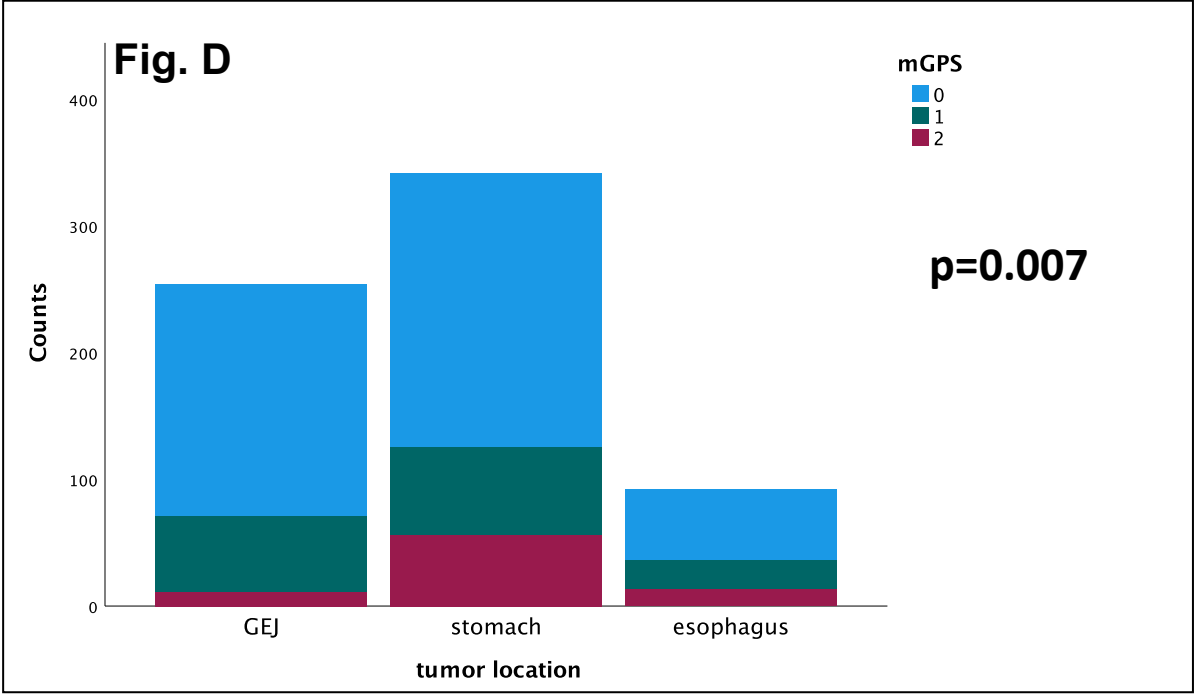

Supplement: Supplementary file 4 — Supplementary Fig. 4: Histograms of modified Glasgow prognostic score (mGPS) according to tumour stage setting (A), weight loss (B) and body mass index (BMI; Fig. 3). P-values estimated with chi-square-method (PDF 80 KB) [file 432_2023_5424_MOESM4_ESM.pdf]

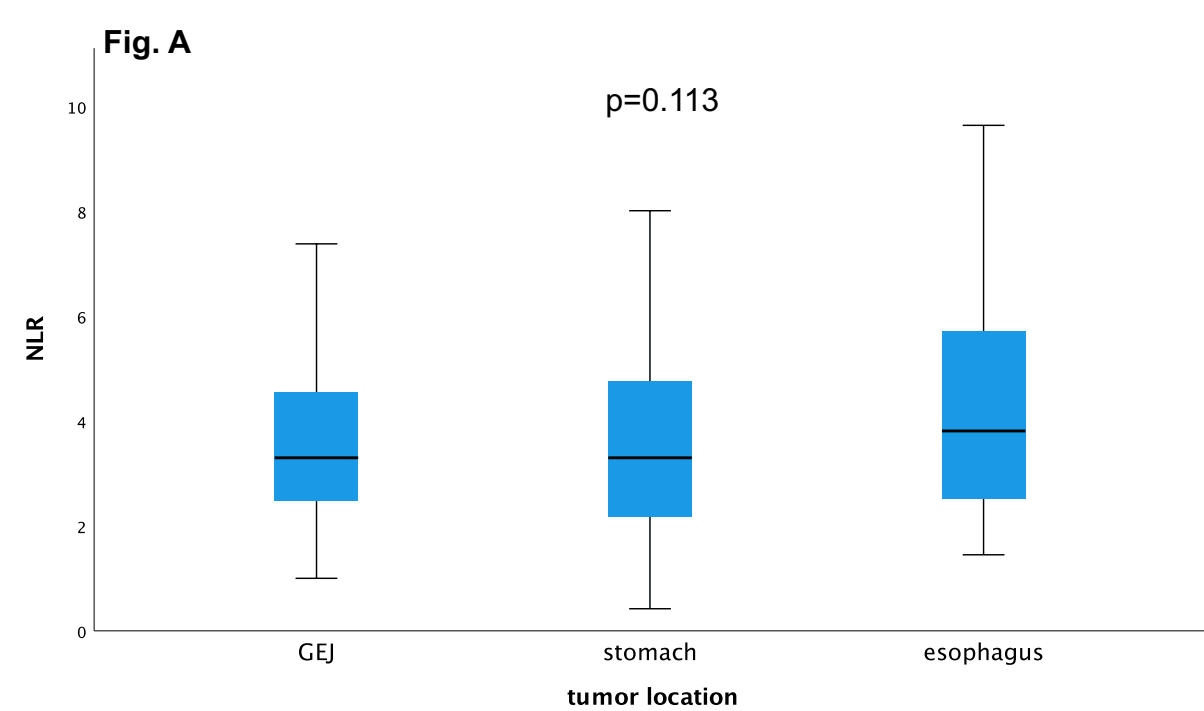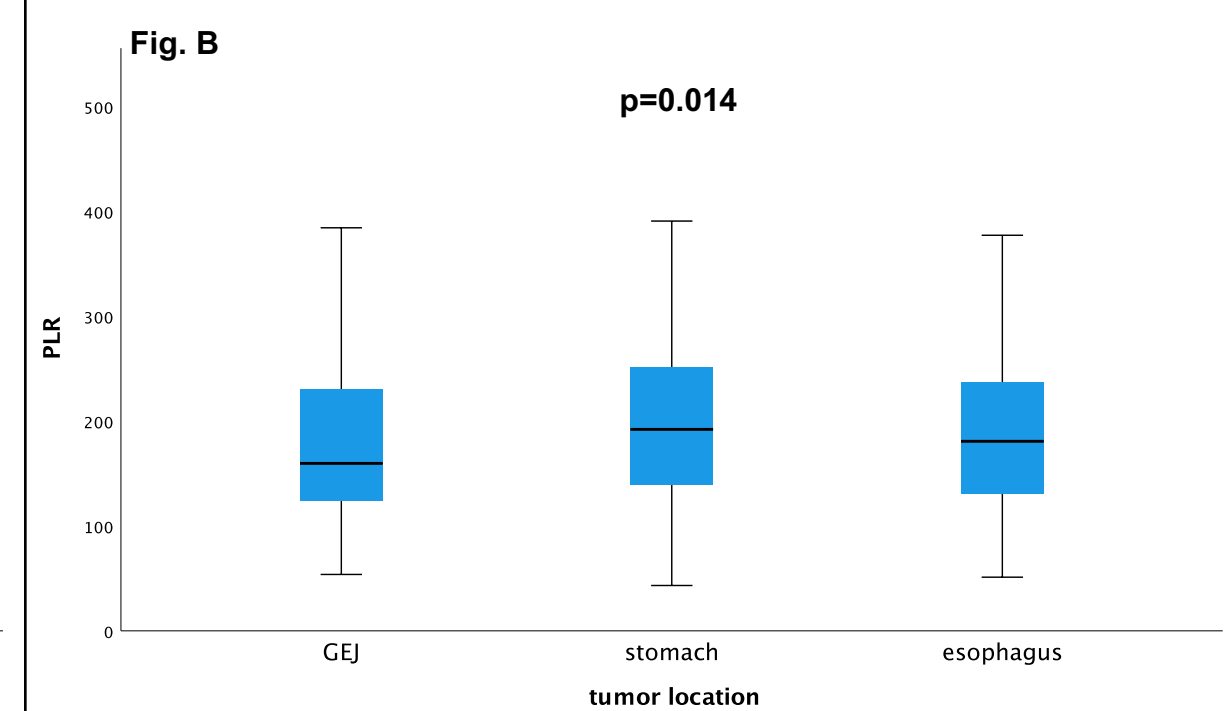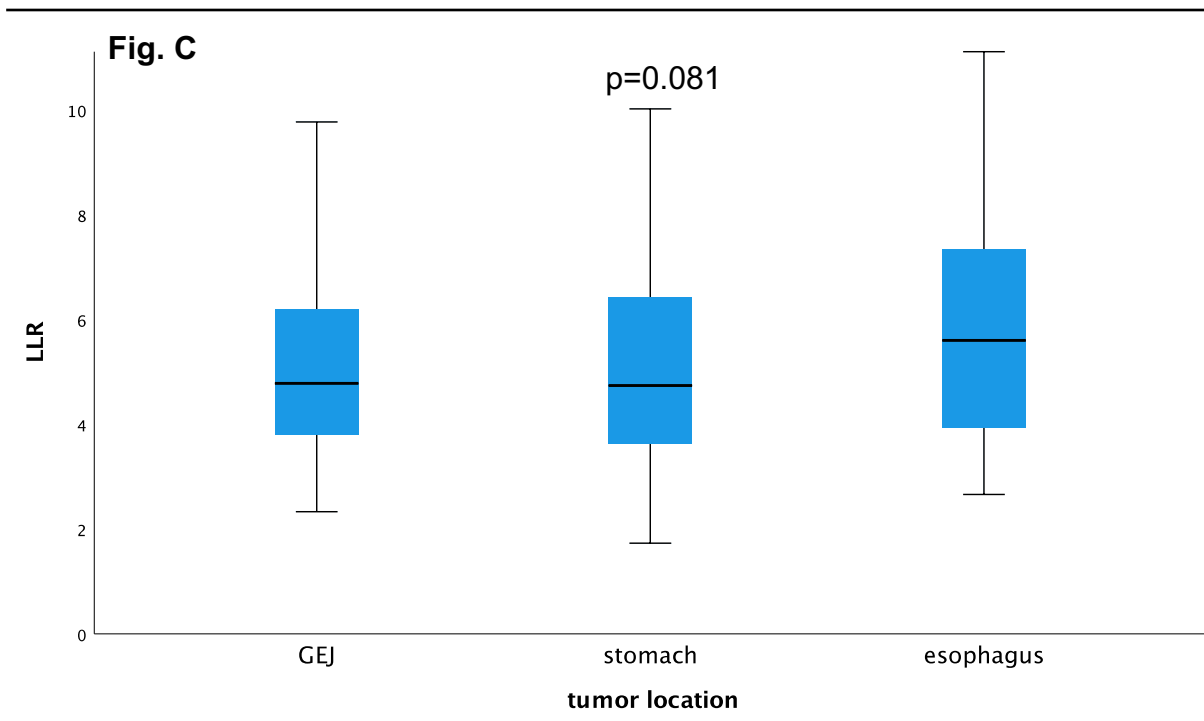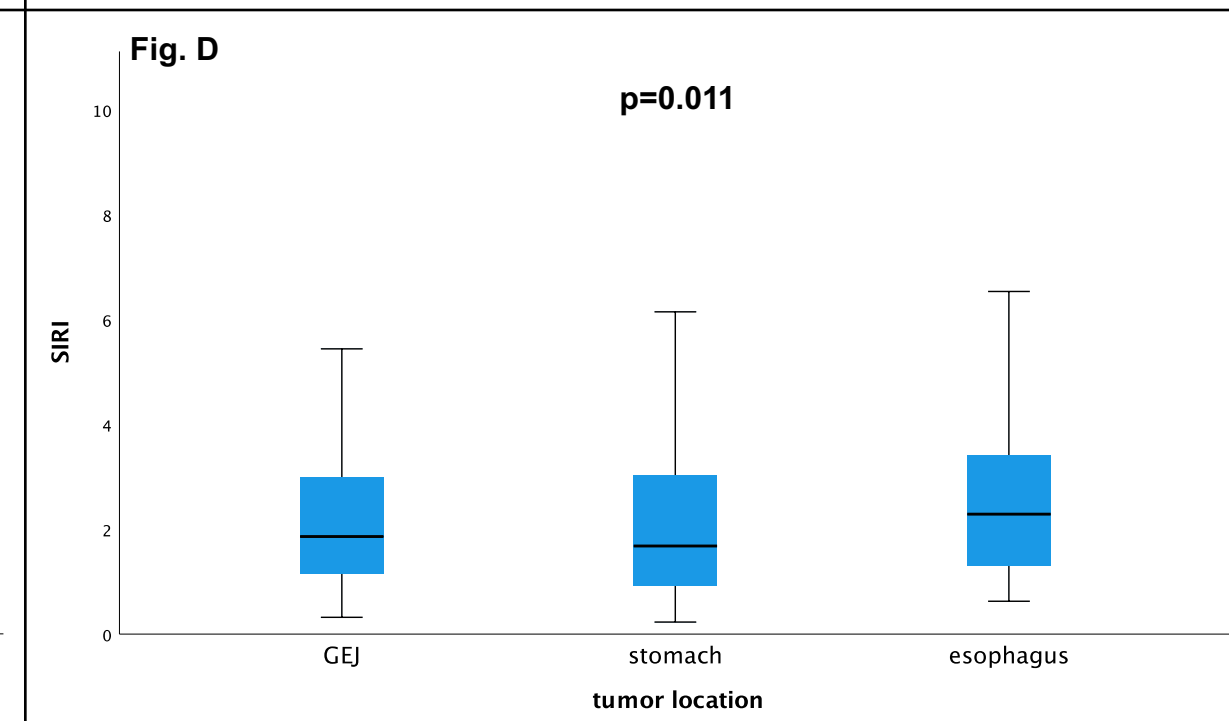

Supplement: Supplementary file 5 — Supplementary Fig. 5: Boxplots of neutrophil-to-lymphocyte ratios (NLR), lymphocyte-to-leucocyte ratios (LLR), platelet-to-lymphocyte ratios (PLR) and systemic inflammation response index (SIRI) according to tumour location (A–D). p-values estimated with Kruskal–Wallis method (PDF 55 KB) [file 432_2023_5424_MOESM5_ESM.pdf]

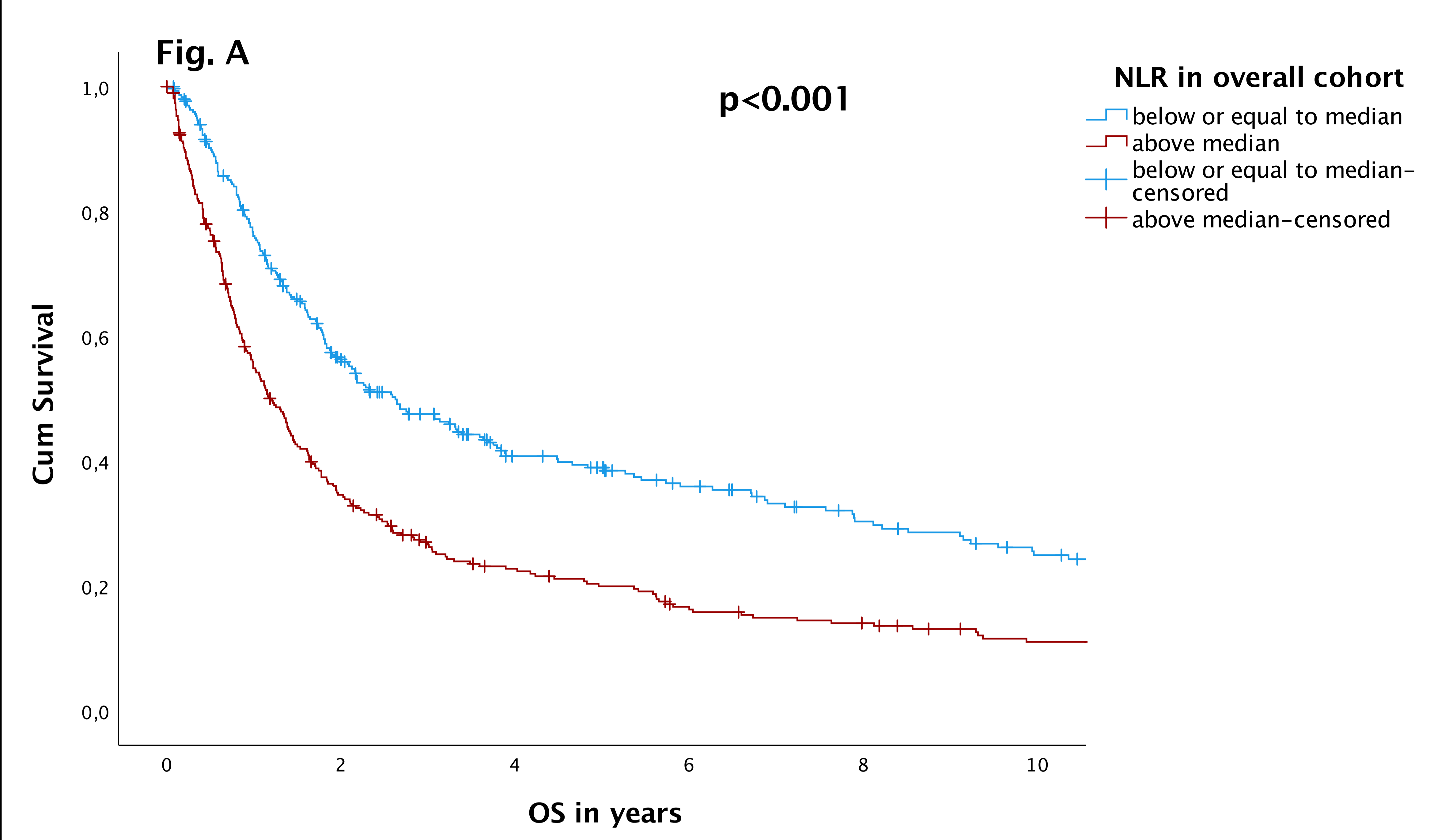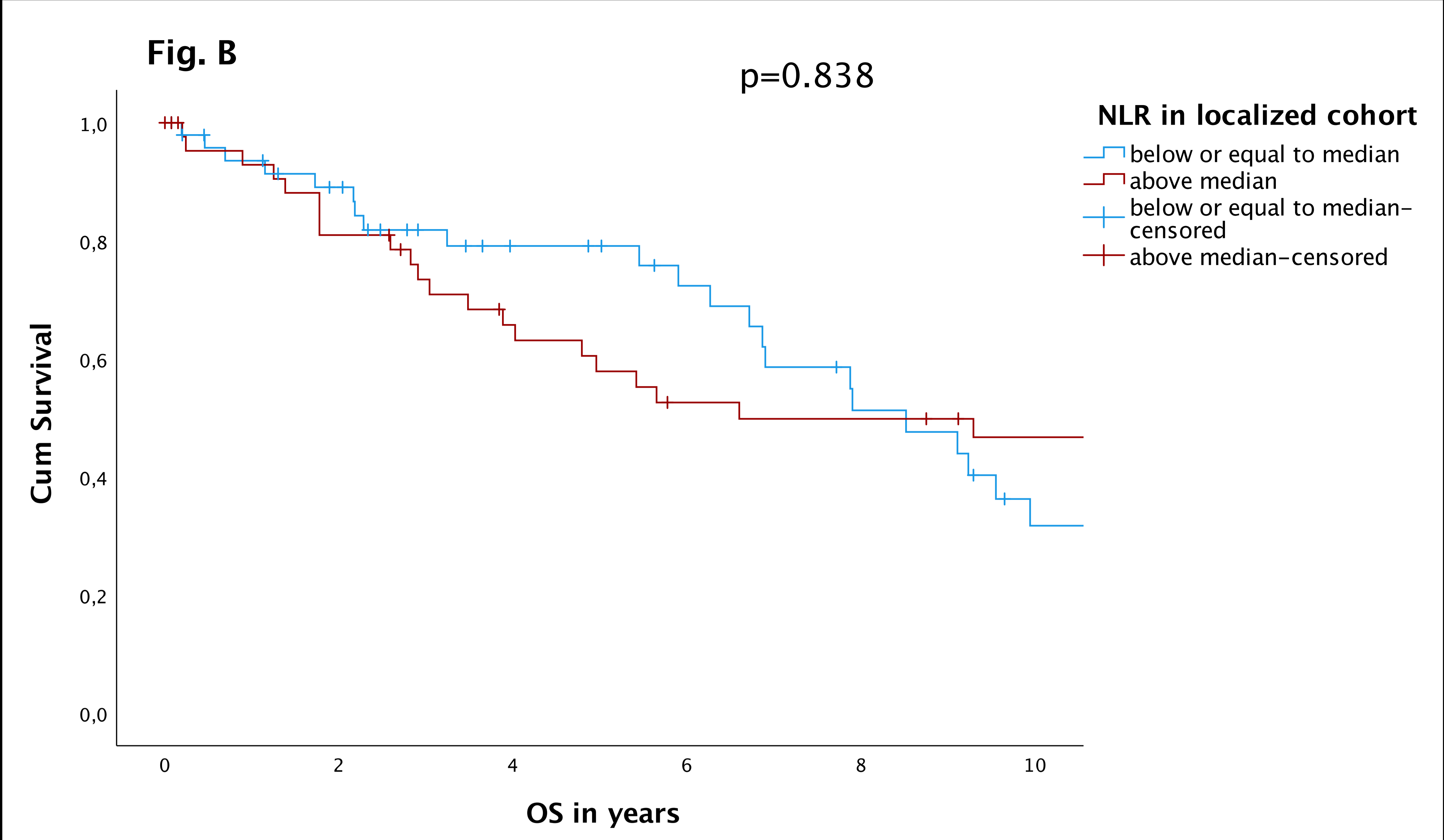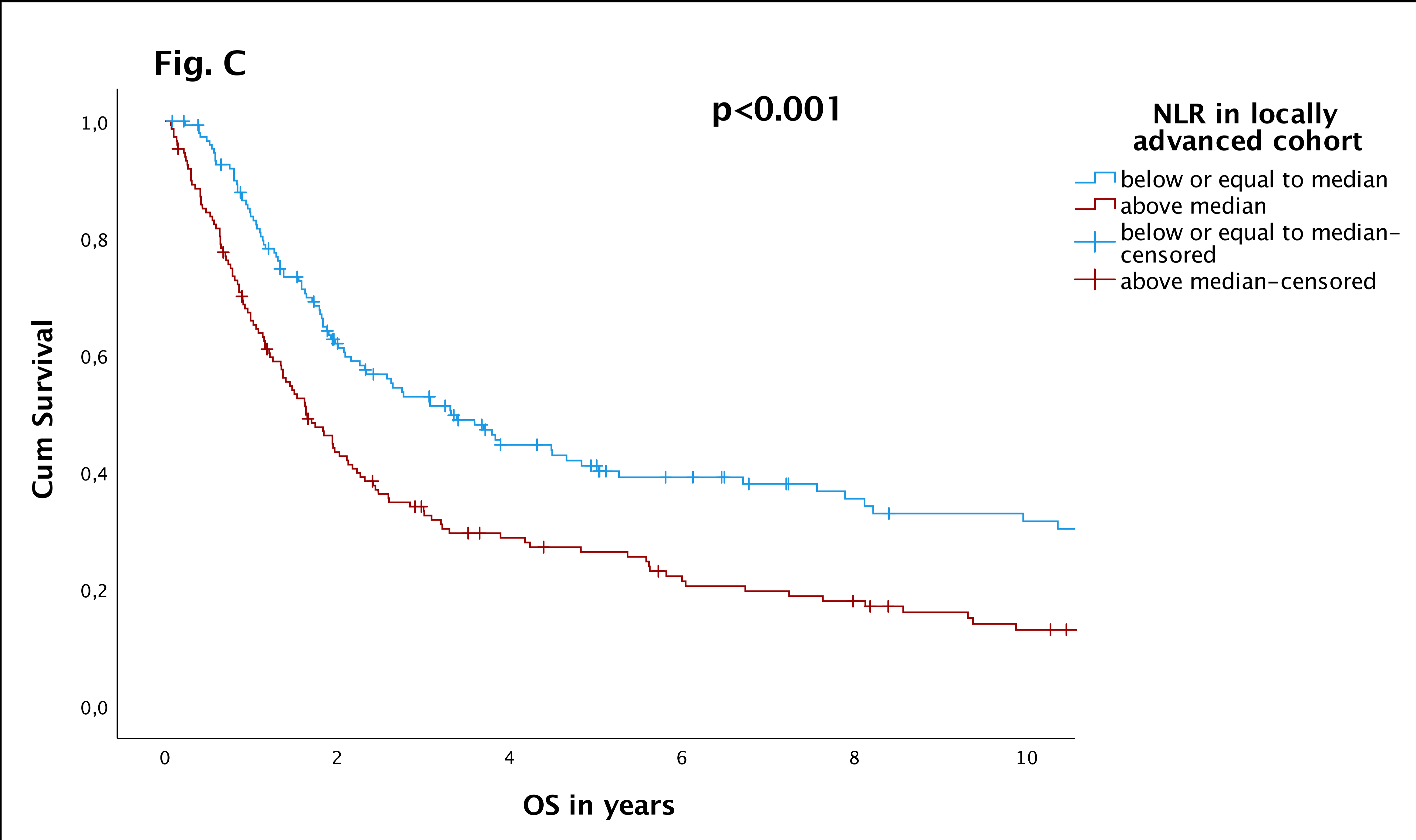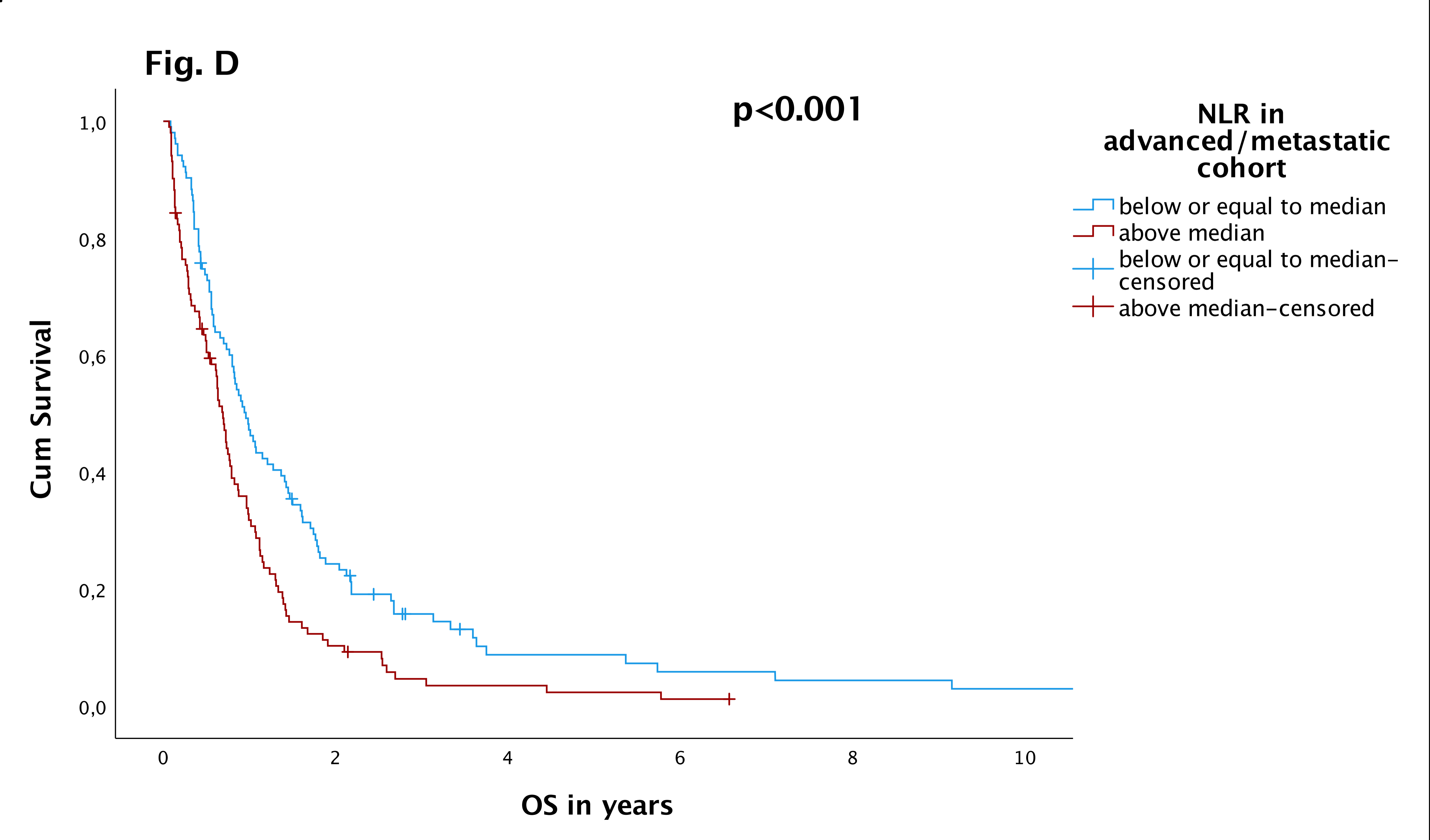

Supplement: Supplementary file 6 — Supplementary Fig. 6: Kaplan–Meier survival estimates of neutrophil-to-lymphocyte ratios (NLR) in association with the overall survival (OS) in a cohort of 769 patients with gastroesophageal adenocarcinoma (A) and sub-cohort of localised (B), locally advanced (C) and advanced or metastatic cancer patients (D). p-values estimated with log-rank test (PDF 153 KB) [file 432_2023_5424_MOESM6_ESM.pdf]

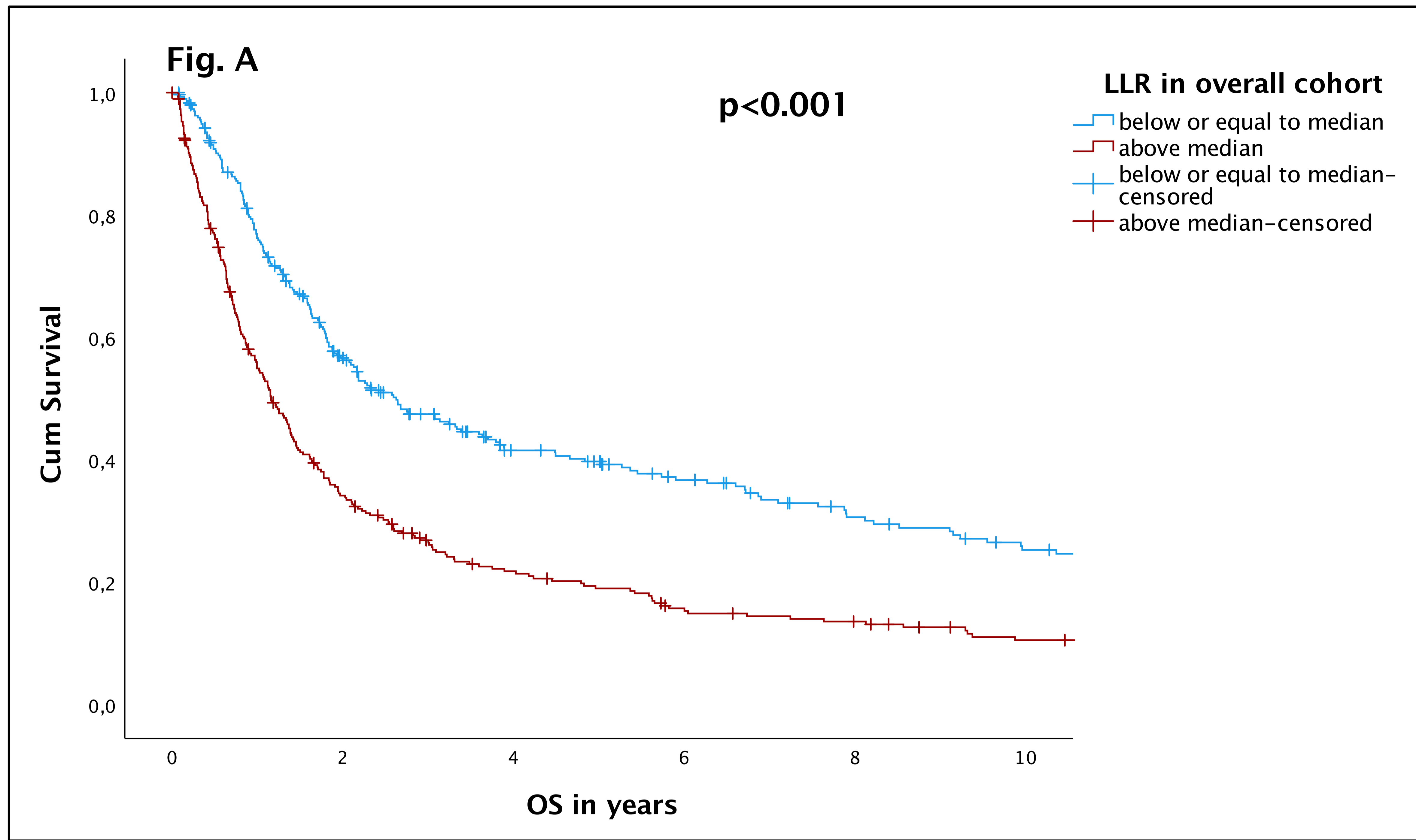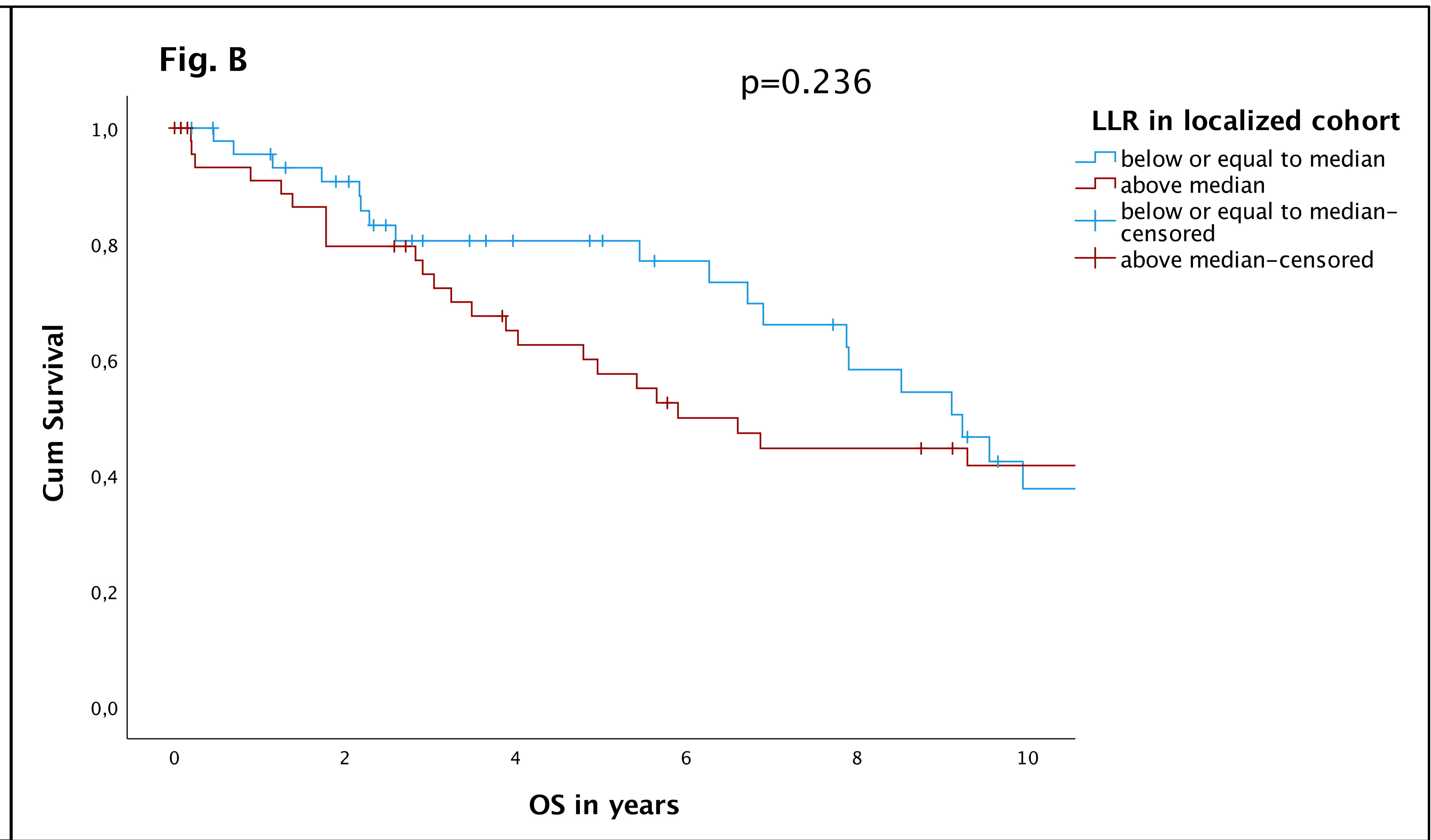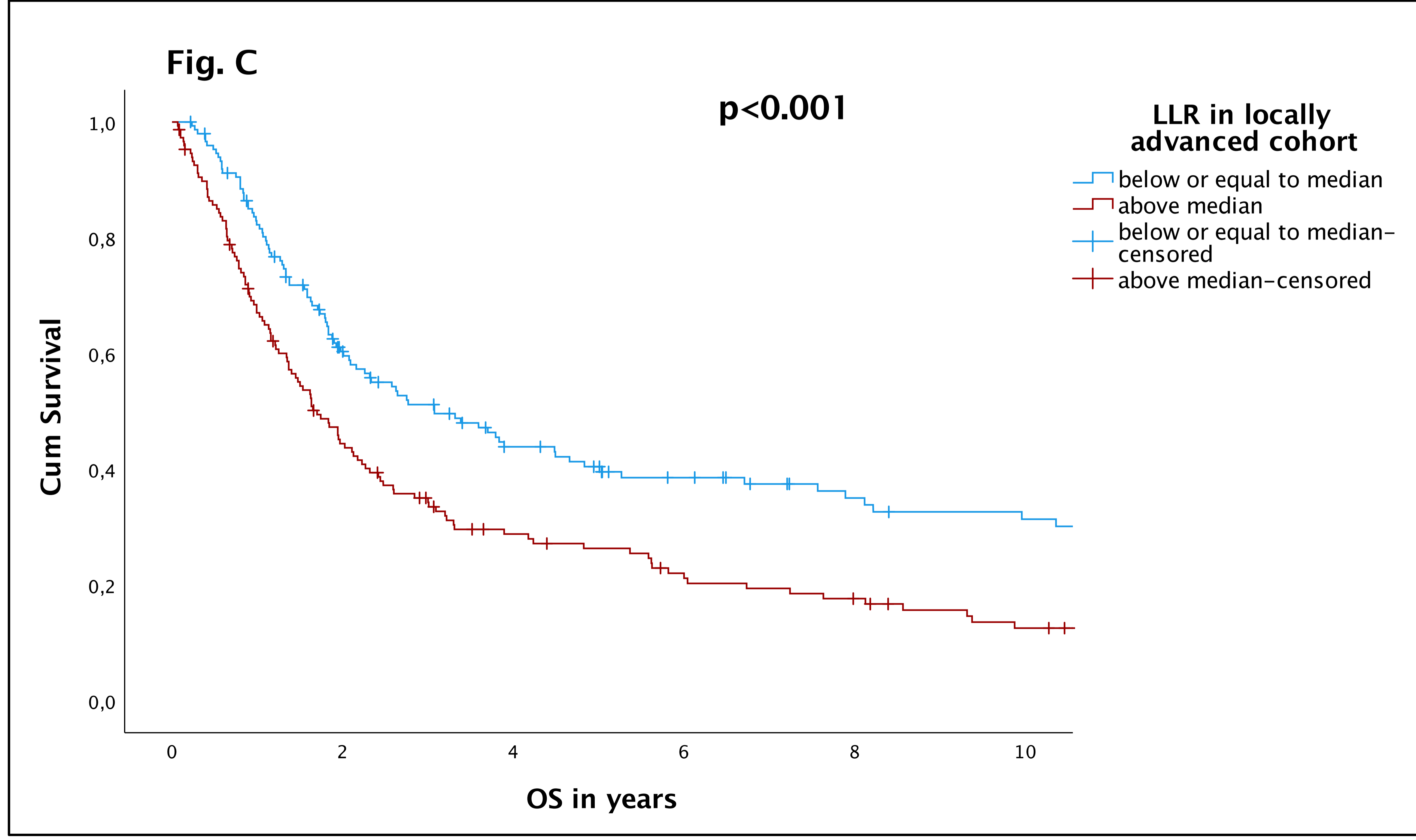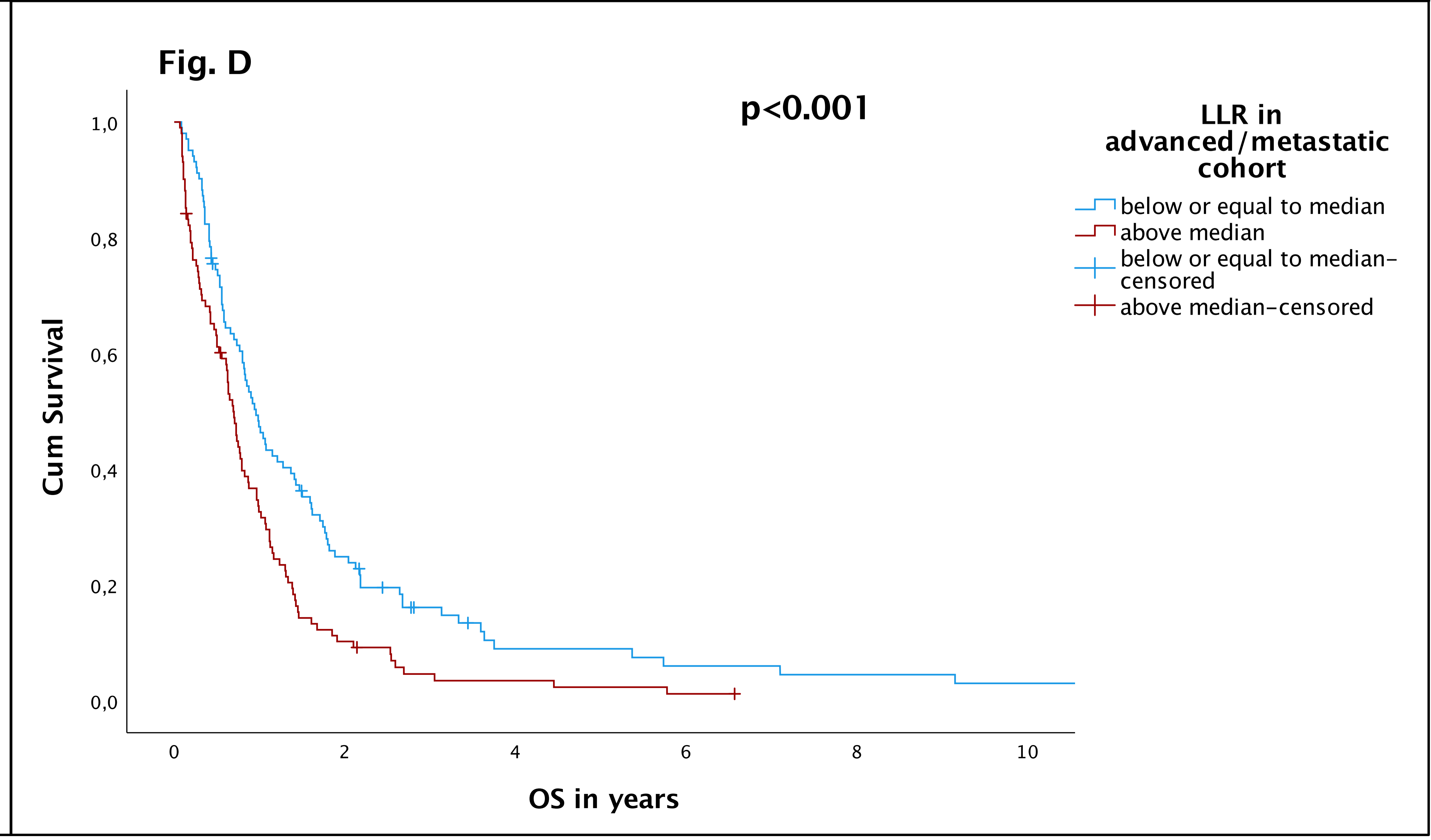

Supplement: Supplementary file 7 — Supplementary Fig. 7: Kaplan–Meier survival estimates of lymphocyte-to-leucocyte ratios (LLR) in association with the overall survival (OS) in a cohort of 769 patients with gastroesophageal adenocarcinoma (A) and sub-cohort of localised (B), locally advanced (C) and advanced or metastatic cancer patients (D). p-values estimated with log-rank test (PDF 153 KB) [file 432_2023_5424_MOESM7_ESM.pdf]

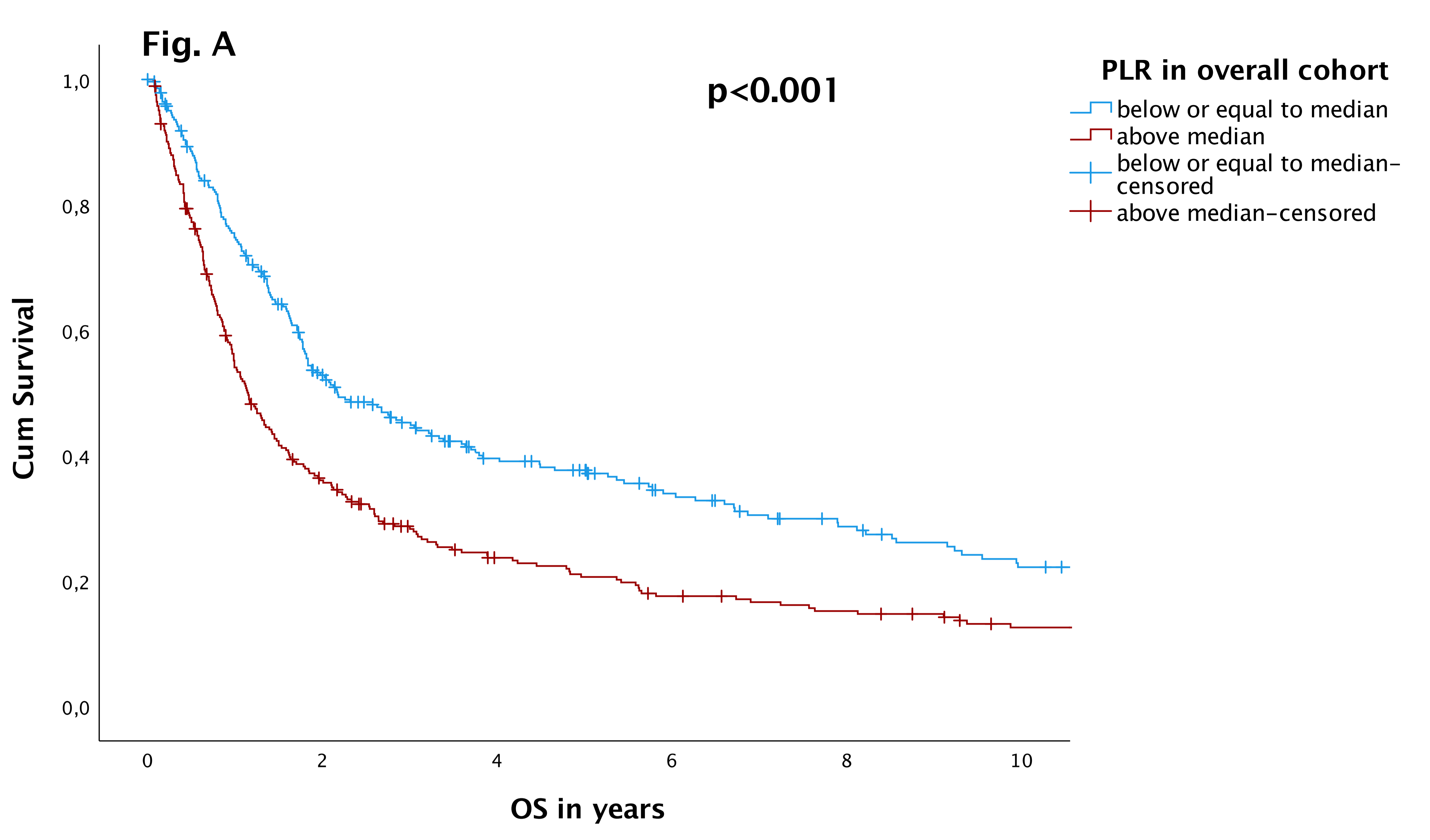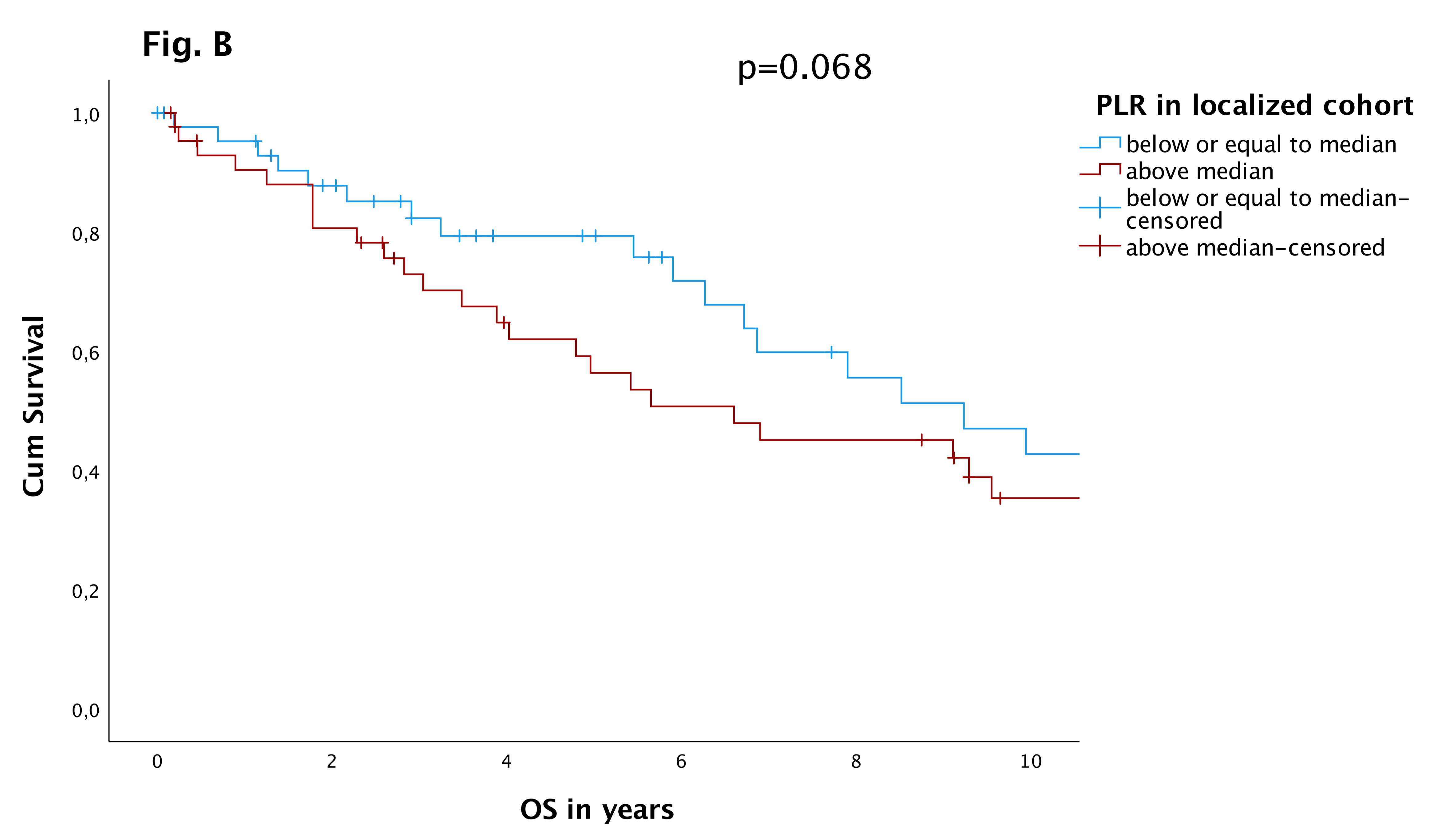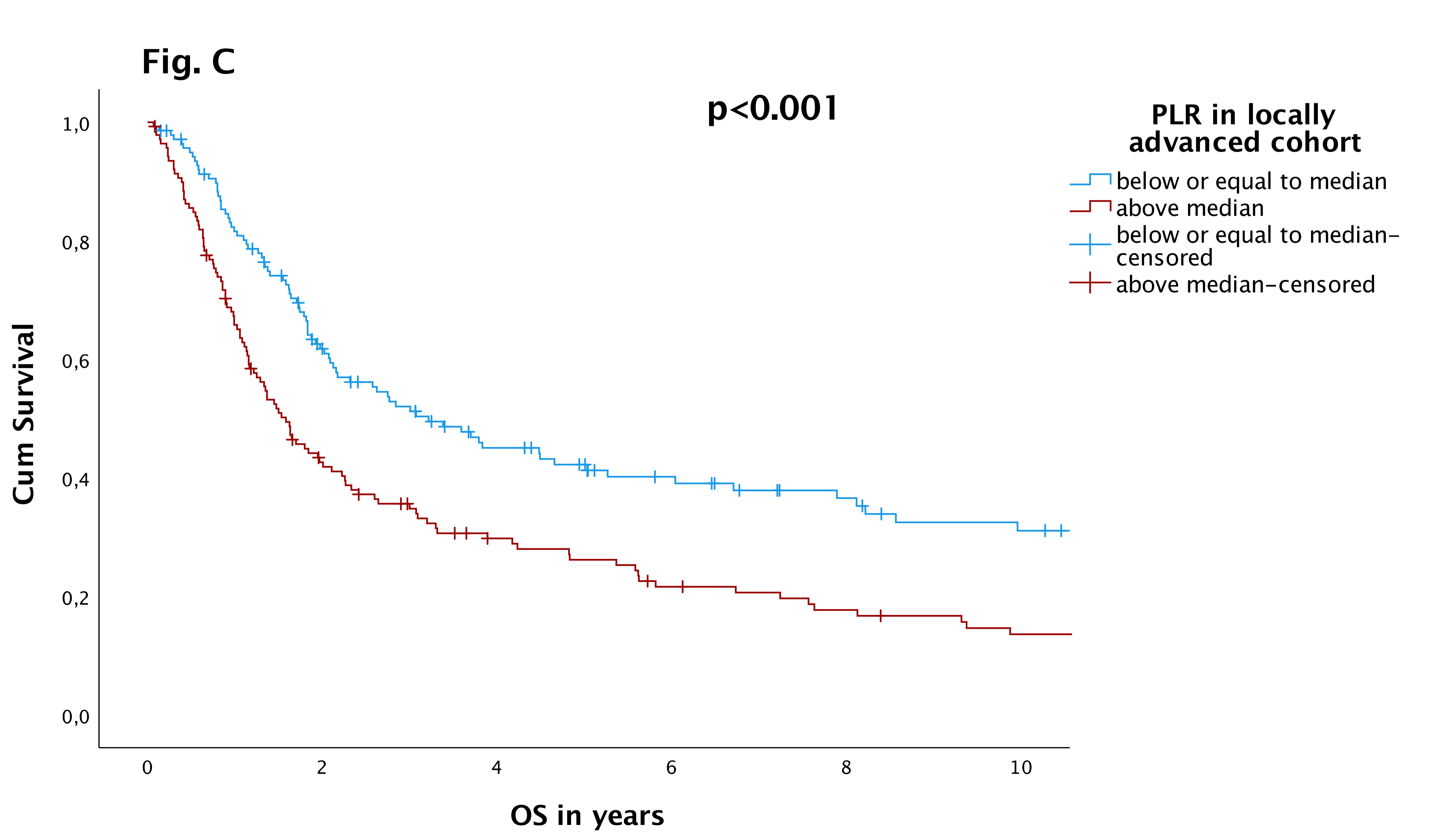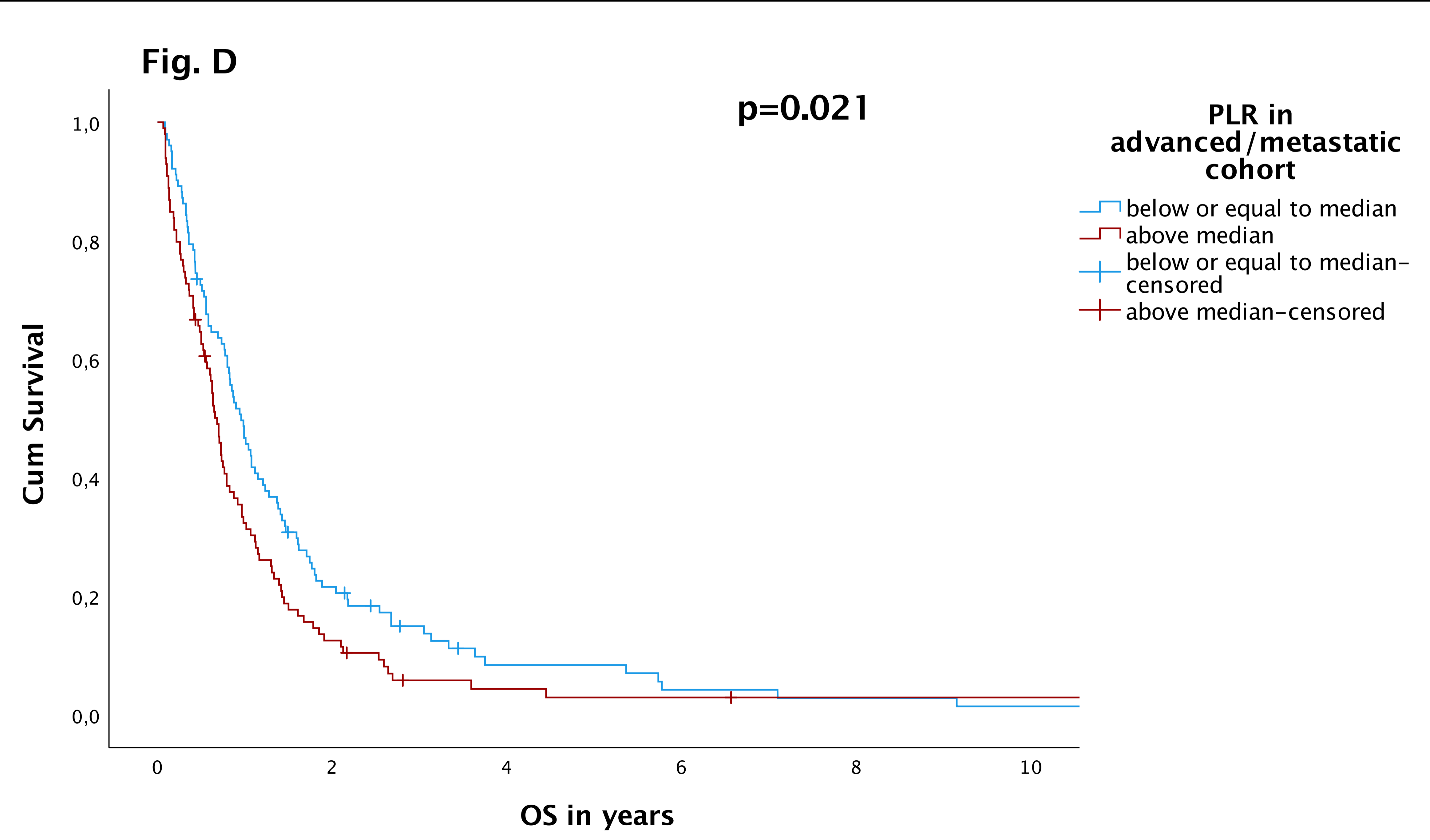

Supplement: Supplementary file 8 — Supplementary Fig. 8: Kaplan–Meier survival estimates of platelet-to-lymphocyte ratios (PLR) in association with the overall survival (OS) in a cohort of 769 patients with gastroesophageal adenocarcinoma (A) and subcohort of localised (B), locally advanced (C) and advanced or metastatic cancer patients (D). p-values estimated with log-rank test (PDF 154 KB) [file 432_2023_5424_MOESM8_ESM.pdf]

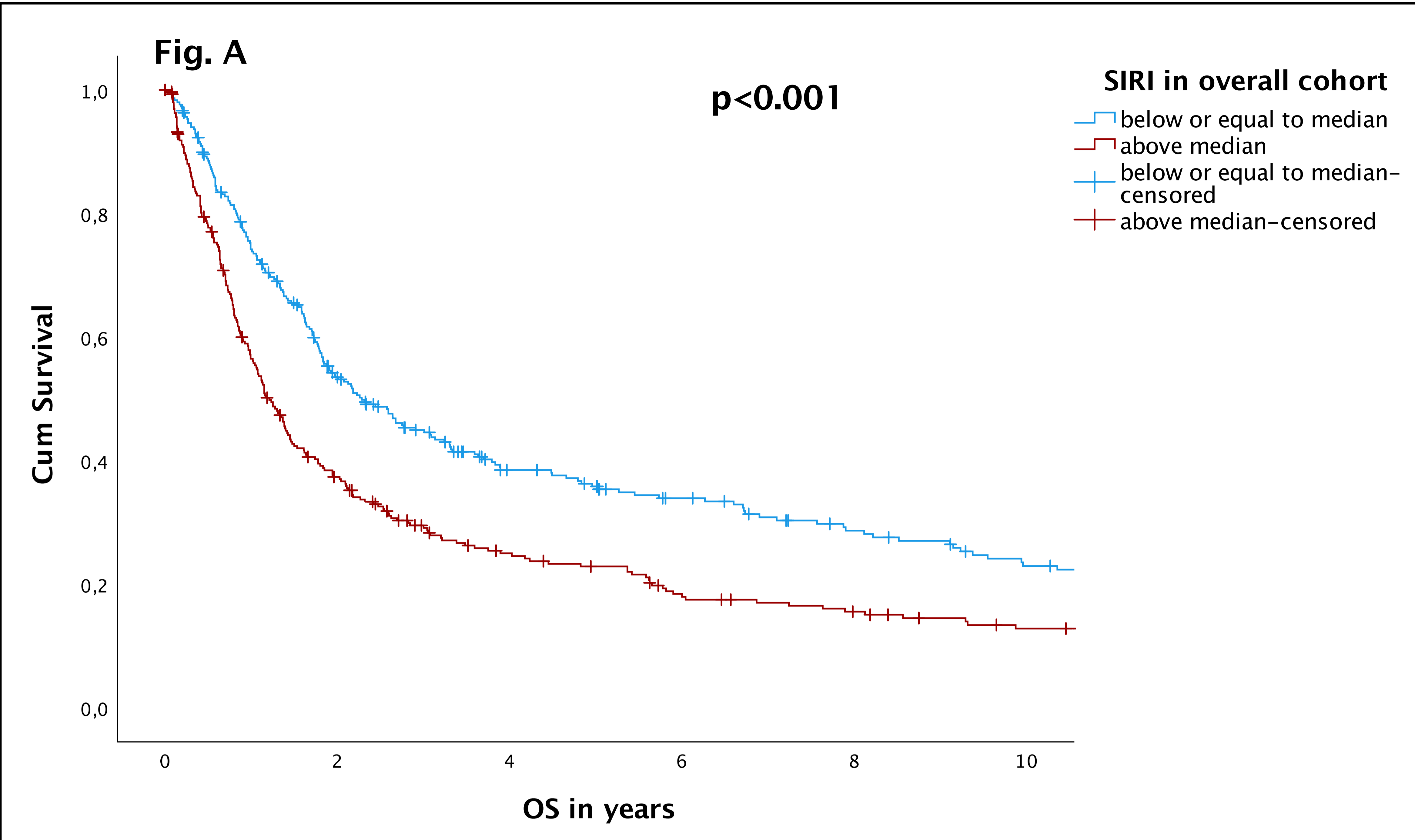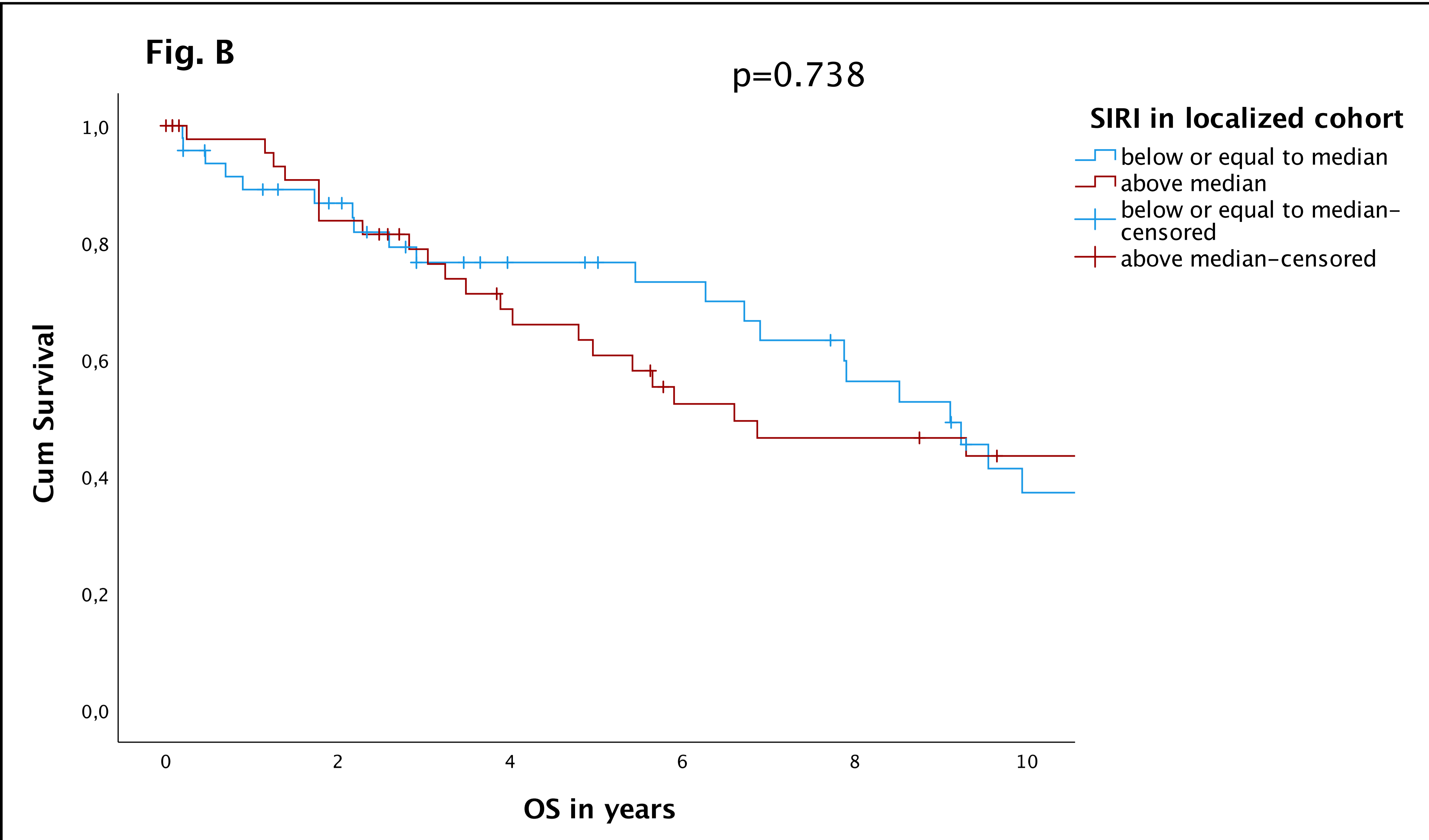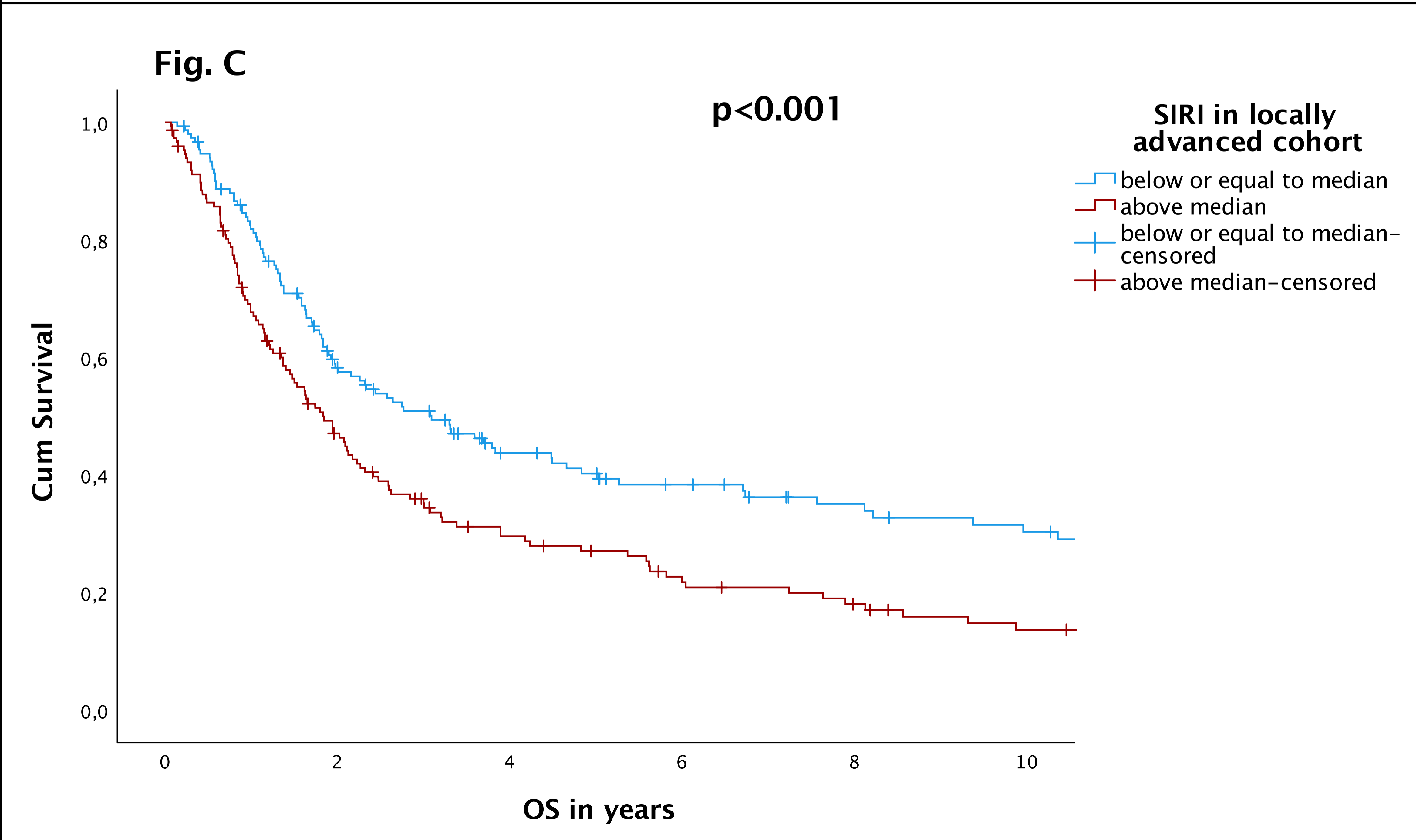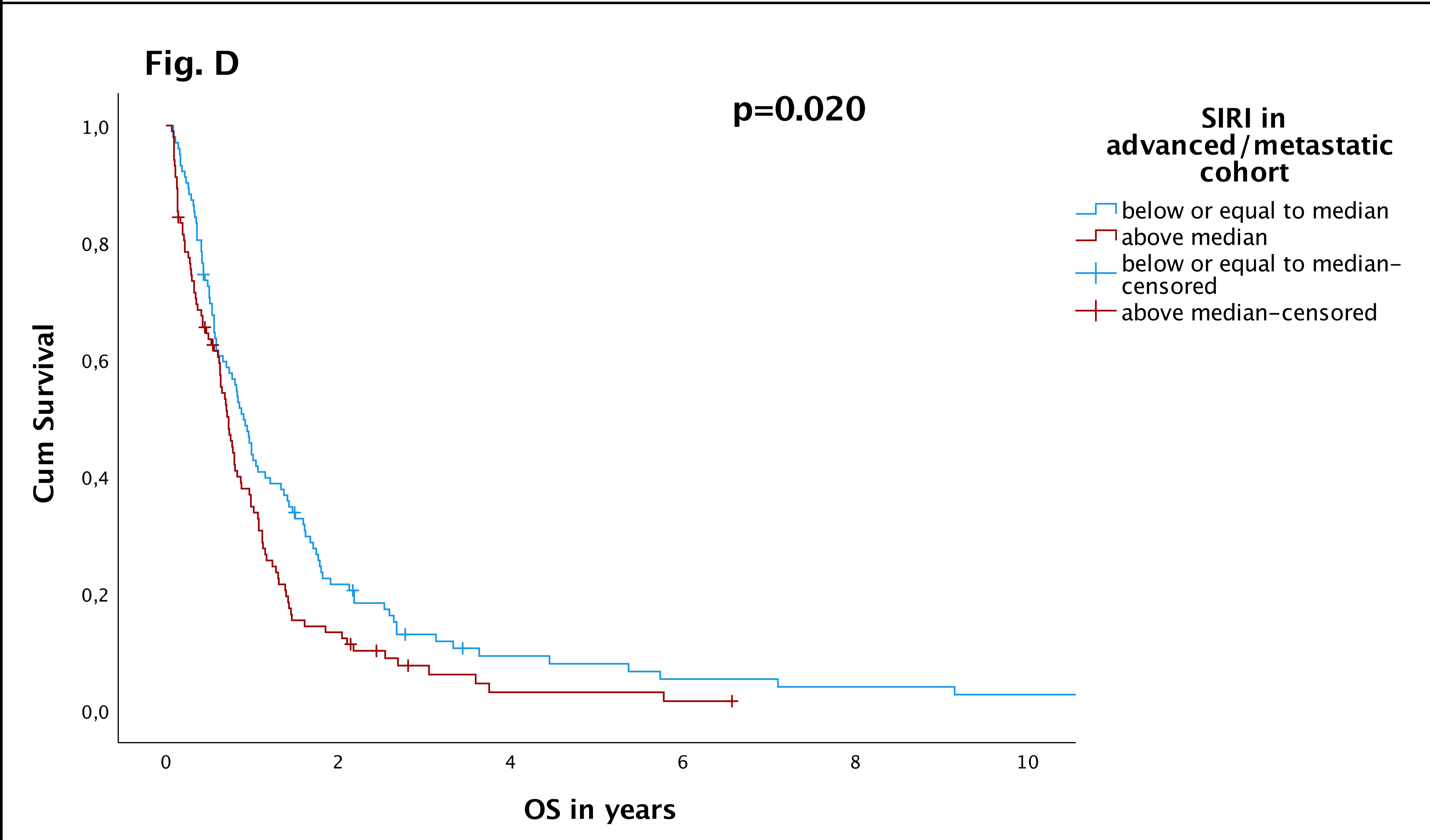

Supplement: Supplementary file 9 — Supplementary Fig. 9: Kaplan–Meier survival estimates of systemic inflammation response index (SIRI) in association with the overall survival (OS) in a cohort of 769 patients with gastroesophageal adenocarcinoma (A) and sub-cohorts of localised (B), locally advanced (C) and advanced or metastatic cancer patients (D). p-values estimated with log-rank test (PDF 155 KB) [file 432_2023_5424_MOESM9_ESM.pdf]
