# Supplementary material for: Systemic inflammatory biomarkers as prognostic tools in patients with gastroesophageal adenocarcinoma
Source: J Cancer Res Clin Oncol. 2023 Sep 26;149(19):17081–91. doi: 10.1007/s00432-023-05424-4 (PMC10657318; doi:10.1007/s00432-023-05424-4)
Supplement: Supplementary file 10 — Supplementary file10 (DOCX 18 KB) [file 432_2023_5424_MOESM10_ESM.docx]

|  | Overall cohort | | | Stage I | | | Stage II+III | | | Stage IV | | |
| --- | --- | --- | --- | --- | --- | --- | --- | --- | --- | --- | --- | --- |
| Variable | n (%) | OS in months (95%CI) | p | n (%) | OS in months (95%CI) | p | n (%) | OS in months (95%CI) | p | n (%) | OS in months (95%CI) | p |
| Sex |  |  | 0.155 |  |  | 0.605 |  |  | 0.092 |  |  | 0.774 |
| male | 524 (68%) | 19.4 (16.9-21.9) |  | 65 (64%) | 111.5 (73.5-149.5) |  | 256 (71%) | 9.9 (7.7-12.1) |  | 203 (66%) | 27.1 (20.0-34.2) |  |
| female | 245 (32%) | 17.4 (13.7-21.1) |  | 36 (36%) | 79.2 (51.9-106.5) |  | 105 (29%) | 9.6 (7.0-12.2) |  | 104 (34%) | 20.5 (15.0-26.0) |  |
| Year of first diagnosis |  |  | **≤0.001** |  |  | 0.078 |  |  | 0.070 |  |  | 0.089 |
| 1990-1995 | 5 (1%) | 31.7 (0-261.1) |  | - | - |  | 3 (1%) | 243.9 (0-583.5) |  | 2 (1%) | 9.8 (n.a.) |  |
| 1996-2000 | 91 (12%) | 27.4 (9.9-44.9) |  | 19 (19%) | 174.8 (38.5-311.1) |  | 59 (16%) | 19.6 (8.3-30.9) |  | 13 (4%) | 5.8 (3.3-8.3) |  |
| 2001-2005 | 139 (18%) | 27.5 (19.4-35.6) |  | 32 (31%) | 79.2 (11.0-147.4) |  | 69 (19%) | 28.0 (17.0-39.0) |  | 38 (12%) | 16.0 (9.0-23.0) |  |
| 2006-2010 | 242 (32%) | 14.0 (11.5-16.5) |  | 24 (24%) | 57.5 (5.9-109.1) |  | 113 (31%) | 20.9 (17.0-24.8) |  | 105 (34%) | 8.2 (6.5-9.9) |  |
| 2011-2015 | 130 (17%) | 15.7 (12.7-18.7) |  | 8 (8%) | 119.3 (n.a.) |  | 51 (14%) | 25.9 (19.0-32.8) |  | 71 (23%) | 10.5 (8.4-12.6) |  |
| 2016-2020 | 162 (21%) | 19.3 (14.8-23.8) |  | 18 (18%) | - |  | 66 (18%) | 46.7 (21.0-72.5) |  | 78 (25%) | 8.7 (4.0-13.4) |  |
| Nicotine |  |  | 0.061 |  |  | 0.062 |  |  | 0.211 |  |  | 0.419 |
| no | 332 (43%) | 17.1 (14.1-20.1) |  | 51 (51%) | 75.2 (18.9-131.5) |  | 145 (40%) | 22.1 (16.4-27.8) |  | 136 (44%) | 9.3 (6.6-12.0) |  |
| yes | 387 (50%) | 20.8 (17.7-23.9) |  | 48 (48%) | 119.3 (47.4-191.2) |  | 190 (53%) | 29.0 (21.0-37.0) |  | 149 (49%) | 10.2 (7.8-12.6) |  |
| missing | 50 (7%) |  |  | 2 (1%) |  |  | 26 (7%) |  |  | 22 (7%) |  |  |
| Alcohol |  |  | 0.107 |  |  | 0.562 |  |  | **0.008** |  |  | 0.593 |
| No or moderate | 632 (82%) | 19.5 (17.5-21.5) |  | 88 (87%) | 110.8 (87.4-134.2) |  | 294 (81%) | 26.7 (20.3-33.1) |  | 250 (81%) | 10.0 (8.3-11.7) |  |
| abuse | 78 (10%) | 14.6 (9.5-19.7) |  | 9 (9%) | 80.6 (41.8-119.4) |  | 36 (10%) | 16.8 (7.5-26.1) |  | 33 (11%) | 7.0 (3.6-10.4) |  |
| missing | 59 (8%) |  |  | 4 (4%) |  |  | 31 (9%) |  |  | 24 (8%) |  |  |
| Weight-loss |  |  | **≤0.001** |  |  | 0.586 |  |  | **0.005** |  |  | 0.112 |
| no | 333 (43%) | 23.6 (18.7-28.5) |  | 59 (58%) | 111.5 (68.9-154.1) |  | 165 (46%) | 31.7 (20.6-42.8) |  | 109 (35%) | 11.0 (8.6-13.4) |  |
| yes | 409 (53%) | 14.6 (12.7-16.5) |  | 39 (39%) | 82.4 (36.3-128.5) |  | 183 (51%) | 19.5 (15.9-23.2) |  | 187 (61%) | 9.2 (7.5-10.9) |  |
| missing | 27 (4%) |  |  | 3 (3%) |  |  | 13 (3%) |  |  | 11 (4%) |  |  |
| Body mass index |  |  | **0.019** |  |  | 0.329 |  |  | 0.285 |  |  | 0.964 |
| <18 | 32 (4%) | 15.0 (2.7-27.3) |  | 2 (2%) | 57.5 (n.a.) |  | 11 (3%) | 19.8 (15.3-24.3) |  | 19 (6%) | 7.0 (5.8-8.2) |  |
| 18-24.9 | 310 (40%) | 17.1 (14.6-19.6) |  | 44 (44%) | 109.3 (72.7-145.9) |  | 137 (38%) | 21.8 (15.5-28.1) |  | 129 (42%) | 9.6 (6.9-12.3) |  |
| >25 | 299 (39%) | 22.2 (18.1-26.3) |  | 45 (45%) | 102.2 (0-223.6) |  | 146 (40%) | 28.0 (15.7-40.3) |  | 108 (35%) | 9.5 (7.2-11.8) |  |
| missing | 128 (17%) |  |  | 10 (9%) |  |  | 67 (19%) |  |  | 51 (17%) |  |  |

Supplementary table 1: Patient characteristics and their association with the overall survival in a cohort of 769 patients with gastroesophageal adenocarcinoma.
