# Supplementary material for: Systemic inflammatory biomarkers as prognostic tools in patients with gastroesophageal adenocarcinoma
Source: J Cancer Res Clin Oncol. 2023 Sep 26;149(19):17081–91. doi: 10.1007/s00432-023-05424-4 (PMC10657318; doi:10.1007/s00432-023-05424-4)
Supplement: Supplementary file 11 — Supplementary file11 (DOCX 18 KB) [file 432_2023_5424_MOESM11_ESM.docx]

|  | overall | | | localized | | | locally advanced | | | advanced or metastatic | | |
| --- | --- | --- | --- | --- | --- | --- | --- | --- | --- | --- | --- | --- |
| Variable | n (%) | OS in months (95%CI) | p | n (%) | OS in months (95%CI) | p | n (%) | OS in months (95%CI) | p | n (%) | OS in months (95%CI) | p |
| Tumorlocation |  |  | 0.432 |  |  | 0.951 |  |  | 0.286 |  |  | 0.120 |
| Gastroesophageal junction | 287 (37%) | 17.5 (14.6-20.4) |  | 23 (23%) | 119.3 (59.1-179.5) |  | 168 (47%) | 22.8 (15.0-30.6) |  | 96 (31%) | 12.0 (10.0-14.0) |  |
| Stomach | 376 (49%) | 19.5 (16.9-22.1) |  | 63 (62%) | 94.8 (59.4-130.2) |  | 139 (38%) | 26.7 (19.3-34.1) |  | 174 (57%) | 9.0 (7.1-10.9) |  |
| Esophagus | 106 (14%) | 16.2 (8.4-24.0) |  | 15 (15%) | 155.4 (28.9-281.9) |  | 54 (15%) | 25.1 (16.0-34.2) |  | 37 (12%) | 7.4 (4.5-10.3) |  |
| Helicobacter pylori |  |  | **0.021** |  |  | 0.897 |  |  | 0.444 |  |  | 0.225 |
| Negative | 282 (37%) | 19.5 (16.6-22.4) |  | 35 (35%) | 110.8. (74.2-147.4) |  | 134 (37%) | 23.1 (15.8-30.4) |  | 113 (37%) | 9.6 (7.5-11.7) |  |
| Positive | 276 (36%) | 23.3 (18.7-27.9) |  | 61 (60%) | 82.8 (49.0-116.6) |  | 125 (35%) | 27.8 (17.8-37.8) |  | 90 (29%) | 11.9 (9.9-13.9) |  |
| missing | 211 (27%) |  |  | 5 (5%) |  |  | 102 (28%) |  |  | 104 (34%) |  |  |
| Lauren |  |  | **0.002** |  |  | 0.140 |  |  | 0.054 |  |  | **≤0.001** |
| Intestinal | 213 (28%) | 25.6 (18.4-32.8) |  | 40 (39%) | 110.8 (53.7-167.9) |  | 107 (30%) | 27.2 (15.6-38.8) |  | 66 (22%) | 14.5 (10.7-18.3) |  |
| Diffuse | 216 (28%) | 17.4 (14.0-20.8) |  | 27 (27%) | 41.8 (24.0-59.6) |  | 96 (27%) | 29.7 (19.8-39.6) |  | 93 (30%) | 7.8 (6.2-9.4) |  |
| Mixed | 23 (3%) | 20.9 (2.7-39.1) |  | 6 (6%) | 94.8 (32.6-157.0) |  | 11 (3%) | 13.8 (11.1-16.5) |  | 6 (2%) | 11.9 (1.7-22.1) |  |
| Missing | 317 (41%) |  |  | 28 (28%) |  |  | 147 (41%) |  |  | 142 (46%) |  |  |
| Her2 positivity |  |  | 0.097 |  |  | 0.629 |  |  | 0.775 |  |  | **0.008** |
| Her2 positive | 67 (9%) | 27.8 (21.8-33.8) |  | 6 (6%) | 119.3 (n.a.) |  | 29 (8%) | 29.0 (15.5-42.5) |  | 32 (10%) | 17.1 (5.5-28.7) |  |
| Her2 negative | 227 (30%) | 16.5 (13.5-19.5) |  | 18 (18%) | n.a. |  | 93 (26%) | 33.0 (16.3-49.7) |  | 116 (38%) | 9.9 (7.6-12.2) |  |
| missing | 475 (62%) |  |  | 77 (76%) |  |  | 239 (66%) |  |  | 159 (52%) |  |  |
| PD-L1 CPS |  |  | 0.643 |  |  | 0.617 |  |  | 0.829 |  |  | **0.020** |
| CPS 0 | 24 (3%) | 20.5 (11.2-29.8) |  | 4 (4%) |  |  | 7 (2%) | 50.1 (6.8-93.4) |  | 13 (4%) | 13.5 (8.8-18.2) |  |
| CPS 1-4 | 7 (1%) | 11.9 (2.9-20.9) |  | 1 (1%) |  |  | 3 (1%) | 11.9 (n.a.) |  | 3 (1%) | 5.4 (0-11.9) |  |
| CPS ≥5 | 20 (3%) | 43.1 (11.6-74.6) |  | - |  |  | 9 (2%) | 46.7 (n.a.) |  | 11 (4%) | 17.0 (1.0-33.0) |  |
| missing | 718 (93%) |  |  | 96 (95%) |  |  | 342 (95%) |  |  | 280 (91%) |  |  |

Supplementary table 2: Tumor characteristics and their association with the overall survival.
