# Supplementary material for: Systemic inflammatory biomarkers as prognostic tools in patients with gastroesophageal adenocarcinoma
Source: J Cancer Res Clin Oncol. 2023 Sep 26;149(19):17081–91. doi: 10.1007/s00432-023-05424-4 (PMC10657318; doi:10.1007/s00432-023-05424-4)
Supplement: Supplementary file 12 — Supplementary file12 (DOCX 16 KB) [file 432_2023_5424_MOESM12_ESM.docx]

|  | **overall** | **localized** | **locally advanced** | **advanced or metastatic** |
| --- | --- | --- | --- | --- |
| **Variable** | **n (%)** | **n (%)** | **n (%)** | **n (%)** |
| White blood cells |  |  |  |  |
| Below normal limit | 16 (2%) | 3 (3%) | 8 (2%) | 5 (1%) |
| Within normal limit | 616 (80%) | 83 (82%) | 300 (83%) | 233 (76%) |
| Above normal limit | 123 (16%) | 14 (14%) | 48 (13%) | 61 (20%) |
| missing | 14 (2%) | 1 (1%) | 5 (2%) | 8 (3%) |
| Neutrophils |  |  |  |  |
| Below normal limit | 12 (2%) | 2 (2%) | 4 (1%) | 6 (2%) |
| Within normal limit | 500 (65%) | 82 (81%) | 256 (71%) | 162 (53%) |
| Above normal limit | 86 (11%) | 10 (10%) | 39 (11%) | 37 (12%) |
| missing | 171 (22%) | 7 (7%) | 62 (17%) | 102 (33%) |
| Lymphocytes |  |  |  |  |
| Below normal limit | 82 (11%) | 10 (10%) | 33 (9%) | 39 (13%) |
| Within normal limit | 513 (67%) | 84 (83%) | 263 (73%) | 166 (54%) |
| Above normal limit | 3 (<1%) | - | 3 (1%) | - |
| missing | 171 (22%) | 7 (7%) | 62 (17%) | 102 (33%) |
| Monocytes |  |  |  |  |
| Within normal limit | 585 (76%) | 92 (91%) | 298 (83%) | 195 (63%) |
| Above normal limit | 12 (2%) | 2 (2%) | 1 (<1%) | 9 (3%) |
| missing | 172 (22%) | 7 (7%) | 62 (17%) | 103 (34%) |
| Eosinophils |  |  |  |  |
| Within normal limit | 569 (74%) | 92 (91%) | 286 (79%) | 191 (62%) |
| Above normal limit | 27 (4%) | 2 (2%) | 13 (4%) | 12 (4%) |
| missing | 173 (22%) | 7 (7%) | 62 (17%) | 104 (34%) |
| Basophils |  |  |  |  |
| Within normal limit | 589 (77%) | 93 (92%) | 297 (82%) | 199 (65%) |
| Above normal limit | 8 (1%) | 1 (1%) | 2 (1%) | 5 (2%) |
| missing | 172 (22%) | 7 (7%) | 62 (17%) | 103 (33%) |
| C-reactive protein |  |  |  |  |
| Within normal limit | 336 (44%) | 61 (60%) | 182 (50%) | 93 (30%) |
| Above normal limit | 382 (50%) | 34 (34%) | 148 (41%) | 200 (65%) |
| missing | 51 (6%) | 6 (6%) | 31 (9%) | 14 (5%) |
| Platelets |  |  |  |  |
| Below normal limit | 34 (4%) | 5 (5%) | 17 (5%) | 12 (4%) |
| Within normal limit | 577 (75%) | 85 (84%) | 283 (78%) | 209 (68%) |
| Above normal limit | 143 (19%) | 10 (10%) | 57 (16%) | 76 (25%) |
| missing | 15 (2%) | 1 (1%) | 4 (1%) | 10 (3%) |
| Albumin |  |  |  |  |
| Below normal limit | 115 (15%) | 8 (8%) | 41 (11%) | 66 (21%) |
| Within normal limit | 548 (71%) | 77 (76%) | 269 (75%) | 202 (66%) |
| missing | 106 (14%) | 16 (16%) | 51 (14%) | 39 (13%) |

Supplementary table 3: Availability of laboratory parameters for the overall cohort and subcohorts
