# Supplementary material for: Systemic inflammatory biomarkers as prognostic tools in patients with gastroesophageal adenocarcinoma
Source: J Cancer Res Clin Oncol. 2023 Sep 26;149(19):17081–91. doi: 10.1007/s00432-023-05424-4 (PMC10657318; doi:10.1007/s00432-023-05424-4)
Supplement: Supplementary file 13 — Supplementary file13 (DOCX 16 KB) [file 432_2023_5424_MOESM13_ESM.docx]

| **variable** | **p-value** | **HR** | **95%CI – lower bound** | **95%CI – upper bound** |
| --- | --- | --- | --- | --- |
| age | 0.3 | 1.009 | 0.992 | 1.025 |
| year of first diagnosis category | 0.744 | 1.022 | 0.897 | 1.165 |
| stage setting | **<.001** | 1.654 | 1.404 | 1.949 |
| BMI | 0.683 | 0.941 | 0.701 | 1.262 |
| weight loss | **0.006** | 1.64 | 1.153 | 2.333 |
| Lauren classification | **0.003** | 1.466 | 1.144 | 1.88 |
| Helicobacter pylori | 0.737 | 1.057 | 0.763 | 1.466 |
| NLR | 0.193 | 1.898 | 0.723 | 4.984 |
| LLR | 0.997 | 0.998 | 0.361 | 2.76 |
| PLR | 0.251 | 0.808 | 0.562 | 1.162 |
| SIRI | 0.511 | 0.854 | 0.533 | 1.368 |
| mGPS | 0.051 | 1.275 | 0.998 | 1.628 |
| treatment | **<.001** | 0.197 | 0.084 | 0.462 |

Supplementary table 4: Multivariate analysis of overall cohort.
