# Supplementary material for: Systemic inflammatory biomarkers as prognostic tools in patients with gastroesophageal adenocarcinoma
Source: J Cancer Res Clin Oncol. 2023 Sep 26;149(19):17081–91. doi: 10.1007/s00432-023-05424-4 (PMC10657318; doi:10.1007/s00432-023-05424-4)
Supplement: Supplementary file 14 — Supplementary file14 (DOCX 15 KB) [file 432_2023_5424_MOESM14_ESM.docx]

| **variable** | **p-value** | **HR** | **95% CI – lower bound** | **95% CI – upper bound** |
| --- | --- | --- | --- | --- |
| age | 0.63 | 1.004 | 0.989 | 1.019 |
| stage | 0.136 | 1.289 | 0.923 | 1.799 |
| alcohol | 0.138 | 1.203 | 0.942 | 1.537 |
| weight loss | **0.007** | 1.58 | 1.136 | 2.197 |
| NLR | 0.997 | 0.997 | 0.133 | 7.467 |
| LLR | 0.94 | 1.081 | 0.143 | 8.15 |
| PLR | 0.289 | 1.222 | 0.844 | 1.769 |
| SIRI | 0.571 | 1.125 | 0.749 | 1.69 |
| mGPS | **0.015** | 1.397 | 1.068 | 1.828 |
| treatment | **<.001** | 0.083 | 0.019 | 0.361 |

Supplementary table 5: Multivariate analysis of patients with locally advanced disease.
